# Factors predicting treatment response to biological and targeted synthetic disease-modifying antirheumatic drugs in psoriatic arthritis – a systematic review and meta-analysis

**DOI:** 10.1007/s10067-024-07193-y

**Published:** 2024-10-28

**Authors:** Tabea Künzler, Manuel Bamert, Haiko Sprott

**Affiliations:** 1 Medical Faculty of the University of Zurich, CH-8006 Zurich, Switzerland; 2 Retail Value Stream, Galenica AG, Untermattweg 8, CH-3027 Bern, Switzerland; 3 Arztpraxis Hottingen, Hottingerstrasse 44, CH-8032 Zurich, Switzerland

**Keywords:** Antirheumatic agents, Artificial intelligence, Meta-Analysis, Precision medicine, Psoriatic arthritis, Systematic review, Treatment outcome

## Abstract

The therapeutic response of patients with psoriatic arthritis (PsA) varies greatly and is often unsatisfactory. Accordingly, it is essential to individualise treatment selection to minimise long-term complications. This study aimed to identify factors that might predict treatment response to biological and targeted synthetic disease-modifying antirheumatic drugs (bDMARDs and tsDMARDs) in patients with PsA and to outline their potential application using artificial intelligence (AI). Five electronic databases were screened to identify relevant studies. A random-effects meta-analysis was performed for factors that were investigated in at least four studies. Finally, 37 studies with a total of 17,042 patients were included. The most frequently investigated predictors in these studies were sex, age, C-reactive protein (CRP), the Health Assessment Questionnaire (HAQ), BMI, and disease duration. The meta-analysis revealed that male sex (odds ratio (OR) = 2.188, 95% confidence interval (CI) = 1.912–2.503) and higher baseline CRP (1.537, 1.111–2.125) were associated with greater treatment response. Older age (0.982, 0.975–0.99), higher baseline HAQ score (0.483, 0.336–0.696), higher baseline DAPSA score (0.789, 0.663–0.938), and higher baseline tender joint count (TJC) (0.97, 0.945–0.996) were negatively correlated with the response to therapy. The other factors were not statistically significant but might be of clinical importance in the context of a complex AI test battery. Further studies are needed to validate these findings and identify novel factors that could guide personalised treatment decisions for PsA patients, in particular in developing AI applications. In accordance with the latest medical developments, decision-support tools based on supervised learning algorithms have been proposed as a clinical application of these predictors.

**Table:** 

**Key messages**
• *Given the often unsatisfactory and unpredictable therapeutic response in patients with Psoriatic Arthritis (PsA), treatment selection must be highly individualized.* • *A systematic literature review was conducted to identify the most reliable predictors of treatment response to biologic and targeted synthetic disease-modifying antirheumatic drugs in PsA patients.* • *The potential integration of these predictors into AI tools for routine clinical practice is discussed.*

**Supplementary Information:**

The online version contains supplementary material available at 10.1007/s10067-024-07193-y.

## Introduction

### Rationale

“Psoriatic arthritis (PsA) is a chronic inflammatory musculoskeletal disease associated with psoriasis” [1]. Today, PsA is considered an independent disease and included in the spectrum of spondyloarthropathies [2, 3].

PsA has a heterogeneous presentation [2], with the majority of patients developing skin symptoms first [1] and arthritis only years later [4].”Wright described five disease patterns that can change over the course of the disease [4, 5]. Hence, assessments should include all possible disease domains, including arthritis, enthesitis, dactylitis, skin and nail disease, and axial involvement [2]. The most important non-musculoskeletal comorbidities are metabolic syndrome, increased cardiovascular risk, uveitis, mental health disorders, and inflammatory bowel disease (IBD) [2, 6–8].

Although the initial description by Moll and Wright suggested a lower level of severity [5], the destructive and progressive nature of the disease, with a similar impact on quality of life (QoL) and functionality as in RA, has been observed [2]. Accordingly, early diagnosis is essential to prevent long-term functional disability [2].

The Classification for Psoriatic Arthritis (CASPAR) criteria are the most widely used diagnostic criteria [9]. For the time after the diagnosis, there is still no agreement regarding the definition of disease activity [1, 10] and no standardised outcome measure to define the declared treatment goals [11]. Treating to remission or low disease activity target, the treat-to-target approach (T2T), is now the current standard strategy of treatment [12]. T2T is supported by evidence of less joint damage, improved QoL and work productivity, and reduced atherosclerosis over time in patients who reach minimal disease activity (MDA) [12].

The antirheumatic drugs approved for PsA include a continuously increasing number of conventional synthetic disease-modifying antirheumatic drugs (csDMARDs), such as methotrexate, sulfasalazine, and leflunomide, and biological drugs (bDMARDs), such as tumor necrosis factor inhibitors (TNFis), interleukin (IL)-12/23 inhibitors, and IL-17A inhibitors, as well as targeted synthetic tsDMARDs, i.e., phosphodiesterase-4 inhibitors (PDE4is) and janus kinase inhibitors (JAKis) [2]. Despite the ongoing development of new drugs, at least 40% of patients fail to respond or have only a partial response [8, 13], and the need for improved therapy remains [11].

Treatment selection among the various options can be challenging because of the heterogeneous and multisystemic presentation and limited head-to-head evidence directly comparing approved medications [1, 14, 15]. Both the European Alliance of Associations for Rheumatology (EULAR) and the ACR suggest a step-up approach with the sequential use of therapeutics [2, 16]. In the 2019 update of the EULAR, nonsteroidal anti-inflammatory drugs (NSAIDs) and local glucocorticoid injections were proposed as initial therapies [16]. In patients with poor prognostic factors and active arthritis, a csDMARD should be rapidly initiated [16]. The requirements and recommendations for starting bDMARDs vary across guidelines and countries [1, 15, 17–19].Individualisation of therapy is crucial because the clinical manifestations of patients with PsA and their response to drugs vary greatly [14, 20, 21]. The current practice involves individualising treatment selection by aligning the most severely impacted domains of the patient with the most reliable evidence regarding drug efficacies for those particular domains [12]. In the event of nonresponse, shifting and cycling through different treatment options are rational consequences [12].

Reliable predictors could optimise treatment selection and minimise both the time to start treatment and the time to the expected response, thereby reducing the risk of further damage and end-organ complications [12]. Despite the promising potential of precision medicine in PsA, there are currently no biomarkers to predict treatment response [12, 22, 23], and thus far, there are no evidence-based strategies to achieve the required individual treatment selection [13].

Artificial intelligence (AI) has been used to study rheumatological diseases for only a few years [24]. Machine learning (ML), as a subset of AI, involves the development of algorithms that enable computers to learn patterns and rules from large amounts of data, allowing them to make predictions or decisions without being explicitly programmed for each task [25]. MLs already show the ability to predict treatment response in other medical fields and even for patients with RA [24, 26]. Supervised learning is one of three types of ML, where machines learn from labelled training data to predict outcomes such as treatment response [25]. In contrast, unsupervised learning classifies similar data on the basis of unlabelled data, and in reinforcement learning, algorithms learn from trial and error. Supervised learning is the most frequently used ML method in medical research [24, 25].

The development of an ML algorithm starts with raw data, so-called Big Data, which in the medical field usually originates from electronic medical records (EMRs) [24]. Big data, including predictors for treatment response, have high dimensionality and a large sample size, which inevitably leads to noise, heterogeneity, false correlations, experimental deviations and statistical bias [24]. EMRs are often incomplete and are based on individual preferences, which makes them difficult to compare [24]. Because ML does not call the input data into question, the performance of the resulting algorithm is directly dependent on the quality of the training data [24]. The next step, known as data preprocessing, is therefore essential to ensure data quality [27]. It comprises data selection, noise filtering, imputation of missing values, restricting the selected data to representative components (feature selection), and finding a common scale of numeric data (normalisation) [27]. The data are usually split into a training dataset to fit the model, a validation dataset to refine it, and a test dataset to evaluate the final model [24]. The complexity and exact challenges that arise in a model for predicting treatment response in PsA remain to be seen.

In the end, the development of successful ML models requires close cooperation between medical and computational experts [27].

### Objectives

The aim of this work was to identify factors that predict treatment response to bDMARDs and tsDMARDs to enable the development of a decision tool for individualised therapy in PsA patients in daily clinical practice. To achieve this objective, a systematic review and meta-analysis of the available literature up to ^25^ October 2023 was performed regarding the success of treatment in light of demographic, biological, and clinical baseline data before the start of treatment. In contrast to previous studies, the quantitative predictive strength of the identified potential predictors of treatment response is also assessed. On the basis of the results, the design of an AI decision support tool was proposed to enable the selection of the most promising therapy for individuals with PsA.

## Methods

This study was conducted according to the Preferred Reporting Items for Systematic Reviews and Meta-Analyses (PRISMA) [28]. A systematic review protocol was developed in advance and can be found in Additional Data S1. It has not been published or registered. Deviations from the protocol and decisions made at a later date are clearly recognizable as such in this work.

### Literature search and selection process

Five electronic databases (Ovid MEDLINE, Embase, the Cochrane Library, Scopus, and Web of Science) were screened. All the articles from database inception until 25th October 2023 were potentially eligible for inclusion. The search was performed via a search protocol with four concepts (PsA, bDMARDs/tsDMARDs, treatment response, and predictors/influencing factors). The full search protocol can be found in Additional Data S2.

**Fig. 1:**
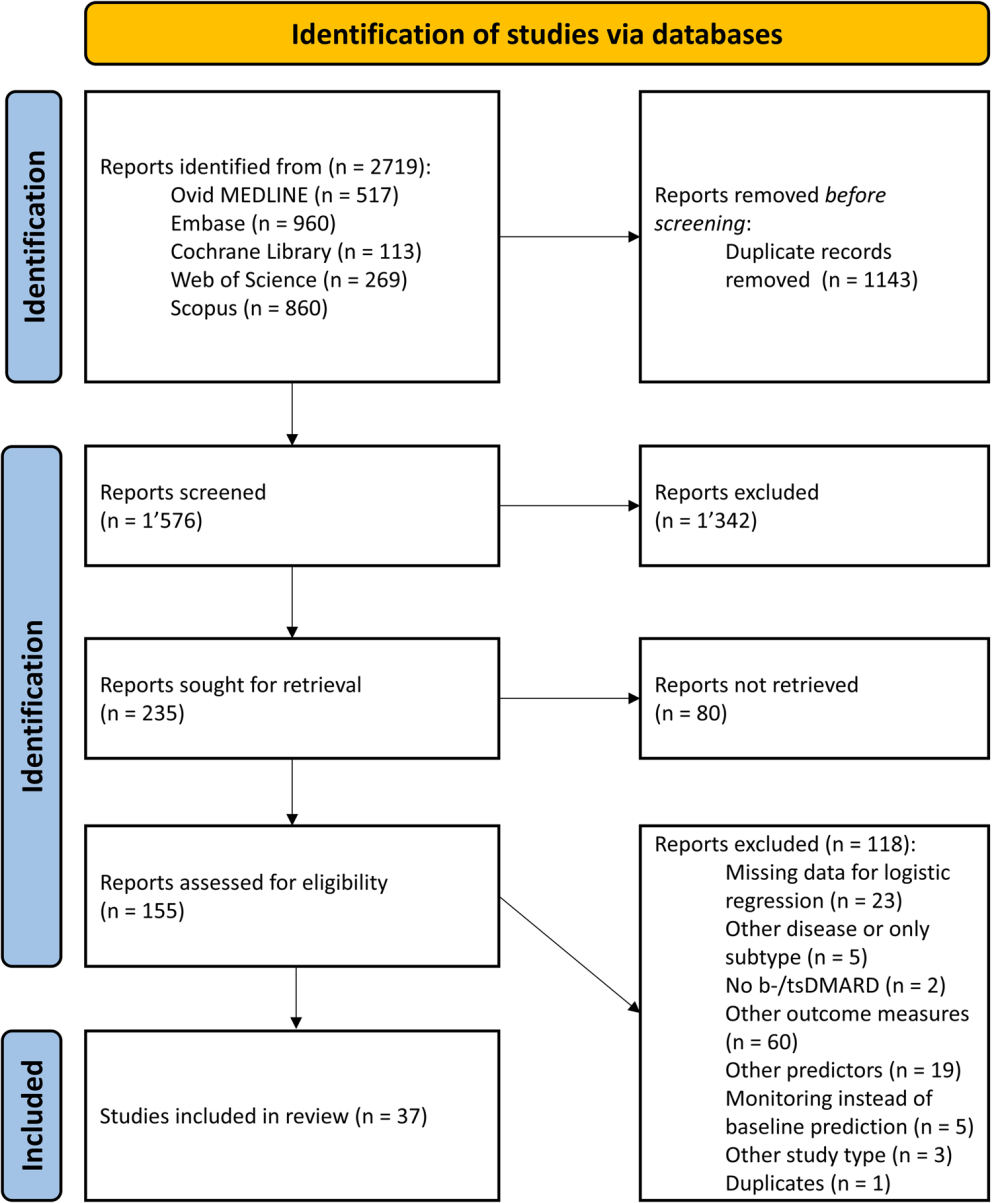
Study Selection Process (PRISMA flowchart). bDMARD = biological disease-modifying antirheumatic drug, tsDMARD = targeted synthetic DMARD

Duplicates were detected automatically by EndNote [29]. Titles and abstracts, and subsequently full texts, were assessed for inclusion. Figure 1 shows the process of article selection in detail. The selection of studies was carried out independently by two reviewers. In the event of discrepancies, a joint discussion and decision were held.

### Eligibility criteria

All studies that were a) available in full text, b) randomised controlled trials (RCTs), case‒control studies or cohort studies, c) human studies, d) included only adults, e) examined bDMARDs or tsDMARDs, f) addressed demographic, clinical or serological baseline markers predicting treatment response in PsA patients, g) used composite measures as outcome measures, and h) contained odds ratios (ORs) or respective raw data to quantify the strength of the suggested predictors were considered for inclusion in this study.

The exact selection of outcome measures was determined after manual screening of the abstracts. It comprises Disease Activity in Psoriatic Arthritis (DAPSA), MDA, EULAR good/moderate response, Composite Psoriatic Disease Activity Index (CPDAI), Psoriatic Arthritis Disease Activity Score (PASDAS), Psoriatic Arthritis Response Criteria (PsARC), ACR response criteria (ACR20/50/70), and Disease Activity Score 28 (DAS28) [30]. They all take into account several disease domains for a global assessment of treatment response [30].

Studies were excluded if they a) examined nonpharmacological therapies or only csDMARDs; b) focused on factors influencing the development of the disease rather than the treatment response; c) were case studies, expert opinions or reviews; d) addressed only psoriasis; d) described monitoring biomarkers measured at a later time point than baseline; e) included only radiological, genetic, synovial, skin, dose/concomitant therapy/drug selection or environmental factors; f) used only single-domain measures, patient-reported outcomes (PROs) or drug survival as outcome measures; g) addressed only the axial disease axPsA; or h) included only PsA-unspecific data from mixed spondyloarthritis (SpA) cohorts.

In addition to the definitive selection of outcome measures, the criteria below have been further refined from the original study protocol. These restrictions were necessary to be able to obtain reliable results and could only be defined after an initial overview of the literature. As detailed in the eligibility criteria, types of predictors were further narrowed down, unspecific SpA data as well as data exclusively about the distinct [31] subset of axSpA were excluded, and odds ratios (ORs) were specified as effect size measures.

### Data items: outcome measures, predictors, effect measure

Full texts containing relevant data were collected, and a spreadsheet recording all the details of the individual studies was generated. This approach allowed the identification of potential predictors and outcome measures that are commonly reported in the literature.

Potential predictors included demographic, clinical, and serological data. Radiological, genetic, synovial, skin, environmental, and therapy-management-related factors were excluded. On the one hand, there are practical reasons for later implementation in the clinic (e.g., synovial biopsy), and on the other hand, there is a lack of markers that have been investigated in more than one study. Although genetic markers have great potential, there are some limitations in the studies published to date that make it difficult to draw firm conclusions in a review [32].

Standardised outcome measures have still not been agreed upon for PsA, which is reflected in the large selection of outcome measures used in earlier publications [31]. Only objectively measurable and reasonably PsA-specific composite measures (DAPSA, MDA, EULAR good/moderate response, CPDAI, PASDAS, PsARC, DAS28, ACR20/50/70) were accepted as outcome measures. As mentioned in the introduction, the selection of outcome measures in PsA remains an unsolved issue [33]. To provide a picture of the response in several disease domains, outcome measures were restricted to so-called composite measures. Single-domain and single-domain recordings of PROs were excluded. The retention rate is considered a representative surrogate measure of treatment effectiveness [31, 34]. However, owing to its dependence on many factors in addition to treatment response (e.g., differing introduction times of drugs [35], prescription guidelines, availability, remission, adherence, adverse events [36–38]) and the correspondingly limited comparability between the studies, drug survival was excluded as an outcome measure. The definition of the outcome measures used can be found in the additional material.

The majority of studies reported the strength of predictors as ORs; therefore, ORs were used as effect size measures in the statistical analysis. Studies reporting statistically incompatible data were excluded from the meta-analysis.

### Data extraction and statistical analysis

A predefined dataset with study characteristics was extracted from each included study. The data included title, author, study size, study type, publication year, mean age, mean disease duration, sex shares, country, outcome measures with measurement times used, and drugs and predictors analysed. Data for the statistical analysis were extracted as ORs of predictor strength with corresponding confidence intervals and *p* values. Whenever possible, ORs from multivariate regression analyses were preferred to those from univariate analyses. If precalculated effect size data were not available, both the ORs and confidence intervals were calculated from the raw data.

The extracted data were grouped by predictor, and if four or more studies had investigated the same predictor, a quantitative meta-analysis was performed. For the others, only a positive or negative correlation was indicated if it was statistically significant. Heterogeneity between studies was quantified by τ^2^, Q, I^2^, and the prediction interval (PI), which gives us a range within which the effects of future studies are expected to lie [39]. Tau squared (τ^2^) estimates the variance in true effect sizes across different studies [39]. The value of Cochran’s Q checks if there is excess variation that cannot be expected from sampling error alone (*p* < 0.05) [39]. Higgins & Thompson’s I^2^ statistic is directly based on the Q value and is defined as the percentage of variability not caused by sampling error [39]. For the majority of predictors, heterogeneity was substantial, and random effects models were used for all meta-analyses via the metagen package in R [39]. The natural logarithms of the ORs and the associated standard errors were calculated as input data for synthesis. To account for multiple reports of the same study about the strength of a predictor across multiple time points and by using multiple composite measures, the weights of those individual reported outcome measures were adjusted to sum to 1 for each study. Within the generated forest plots, the studies were sorted according to the time at which the treatment response was assessed.

Sensitivity analyses were performed for predictors with at least 10 data points (K ≥ 10). Outliers as well as influential data points were identified and excluded, and subgroup analyses were carried out on the basis of the medication and outcome measures used [39]. The study results were identified as outliers by the find.outliers function of the dmetar package if the confidence intervals of their effect sizes did not overlap with the confidence interval of the pooled effect size [39]. Influential data points were searched for via the leave-one-out method [39]. The influence analysis function of the dmetar package allows the adjusted ORs to be displayed as forest plots sorted by effect size or heterogeneity [39]. Subgroup analyses were performed via the update.meta function [39]. Owing to the expected varying treatment effects of the different drugs and the expected differences in the assessment of treatment response depending on the outcome measure used, independent estimates of τ^2^ were assumed for the subgroups and used in the calculations.

### Study risk of bias assessment

The risk of bias assessment of the included observational studies was performed via the Newcastle‒Ottawa Scale (NOS) [40]. This nine-star system includes evaluation of the following parameters: representativeness of the exposed cohort, selection of the nonexposed cohort, ascertainment of exposure, outcome of interest at the start of the study, comparability of cohorts, assessment of outcomes, follow-up duration, and adequacy of follow-up. RCTs were assessed via the Risk of Bias 2 (RoB 2) tool [41]. The completed assessment forms, including the domains comprised in RoB 2, can be found in Additional Tables S1-S2. There was no weighting or exclusion based on the assessment, but it was taken into account for the overall discussion of the results.

### Reporting bias assessment

Publication bias was tested by generating and visually assessing contour-enhanced forest plots and by performing Egger regression (t value) as well as Begg and Mazumdar correlation (z value) tests [42]. The calculations were only carried out for predictors with at least 10 data points (K ≥ 10). The results were incorporated into the certainty assessment described below. For those studies with evidence of publication bias, corrected pooled ORs were calculated via the Duval & Tweedie trim and fill method [39].

### Certainty assessment

The certainty of evidence (CoE) of each statistically analysed predictor was assessed via the GRADE approach. GRADE describes certainty as very low, low, moderate or high [43]. The variablesconsidered can be found in the additional material [43].

## Results

### Description of the studies

The database search initially identified 2,719 records (Ovid MEDLINE = 517, Embase = 960, Cochrane Library = 113, Web of Science = 269, Scopus = 860). After exclusion of duplicates, 1,576 records were manually screened by title and abstract. Eighty of the remaining 235 articles were excluded because they were not available as full-text versions (conference abstracts or lack of respective licences by the University of Zurich). Finally, 37 of the 155 studies included in the full-text screening met the eligibility criteria, and their data were extracted (for a detailed PRISMA flowchart, see Fig. 1). The included studies reported data from 17,042 patients with PsA. These patients had a median age of 48.0 years, a median disease duration of 7.85 years, and a mean proportion of men (46.3%). Their individual characteristics are shown in Table 1.

**Table 1:** Characteristics of included studies

Reference	Study size	Mean age^a^ (years) (*)	Male^a^ (%)	Mean disease duration^ab^ (years) (*)	Drugs	Outcome measure	Timepoints of outcome measurements (weeks)	Country	Study design	NOS score (RoB 2)
Chimenti et al. (2017) [44]	221	55	43.4	12	TNFi	DAS28, CPDAI, DAPSA, MDA	22, 54, 102	Italy	Prospective, single-center, cohort study	7
Venerito et al. (2022) [45]	119	52.7	44.5	7.4	SEC	DAPSA	52	Italy	Retrospective, multicenter, cohort study	7
Au et al. (2014) [46]	390	47.0	62.8	NR	IFX, GOL	ACR	14	International	Retrospective, multicenter, RCT	(some concerns)
Hojgaard et al. (2015) [47]	1′388	48*	51.1	4*	ADA, ETA, IFX	EULAR response, ACR	26	Denmark	Retrospective, multicenter, cohort study	7
Mease et al. (2022) [48]	175	NR	NR	NR	APT	cDAPSA	52	USA	Retrospective, multicenter, RCT	(low)
Iannone et al. (2013) [49]	135	53.2	50.4	10.0	ADA, ETA, IFX	DAS28	varying	Italy	Retrospective, multicenter, cohort study	9
Glintborg et al. (2013) [50]	548	48*	52.4	7*	ADA, ETA, IFX, GOL	ACR, EULAR response	13–26	Denmark	Prospective, multicenter, cohort study	7
Iannone et al. (2020) [51]	238	49.2	47.1	4.5	ADA, ETA, IFX, GOL, CZP, UST, SEC, APT	MDA	52	Italy	Retrospective, single-center, cohort study	7
Chimenti et al. (2012) [44]	85	48.7	49.1	6.5	ADA, ETA	EULAR response	22	Italy	Prospective, single-center, cohort study	9
Michelsen et al. (2017) [52]	3′971	48.3	51.6	1.3*	TNFi	DAS28, EULAR response, DAPSA	13	Norway	Prospective, multicenter, cohort study	6
Michelsen et al. (2017) [53]	2′054	69.9	48.9	0.8*	TNFi	MDA	13	Norway	Prospective, multicenter, cohort study	6
Behrens et al. (2018) [54]	1′684	50.0	49.4	9.5	ADA	MDA	26	Germany	Retrospective, multicenter, cohort study	7
Hojgaard et al. (2016) [55]	1′943	48.0	46.4	4*	ADA, IFX, ETA	EULAR response, ACR	26	Iceland, Denmark	Retrospective, multicenter, cohort study	7
Hojgaard et al. (2016) [55]	69	50.4	43.5	1.4*	cs-/ bDMARDs	ACR, MDA, DAPSA	17	Denmark	Prospective, multicenter, cohort study	7
Mease et al. (2020) [56]	424	50.4	45	NR	ABT	ACR20	24	International	Prospective, multicenter, RCT	(low)
Gratacos et al. (2007) [57]	69	42.5	39.1	8	IFX	ACR50	38	Spain	Prospective, multicenter, cohort study	8
Iervolino et al. (2012) [58]	136	45.6	42.6	5.2	TNFi	MDA	13	Italy	Prospective, single-center, cohort study	7
Kristensen et al. (2016) [59]	274	47*	41.6	7.7*	ADA, ETA, IFX, GOL, CZP	ACR	13	Sweden	Prospective, multicenter, cohort study	7
Ramonda et al. (2021) [60]	608	52.8	41.3	9.5	SEC	EULAR response	26	Italy	Prospective, multicenter, cohort study	8
Van den Bosch et al. (2010) [61]	442	47.8*	50.0	10.6	ADA	ACR, EULAR response	12	International	Prospective, multicenter, cohort study	8
Smolen et al. (2021) [62]	868	49.8	44.4	6.8	TNFi, UST	MDA, cDAPSA	26	International	Prospective multicenter, cohort study	7
Saad et al. (2010) [63]	596	45.7	47.0	12.4	ADA, ETA, IFX	EULAR response, DAS28	26	United Kingdom	Prospective, multicenter, cohort study	6
Hojgaard et al. (2018) [64]	1′750	47.9	46.6	3*	ADA, ETA, IFX, GOL, CZP	EULAR response, ACR	13, 26	Denmark	Prospective, multicenter, cohort study	6
Miyagawa et al. (2022) [65]	81	54.3	51.9	5.9*	IL-17i, TNFi	MDA, DAPSA	52	Japan	Retrospective, single-center, cohort study	6
Ogdie et al. (2023) [66]	84	43.3	46.3	4.2	SEC	DAPSA	12	International	Prospective, multicenter, RCT	(some concerns)
Wagner et al. (2013) [67]	405	47*	60.2	NR	GOL	ACR, DAS28	14	International	Prospective, multicenter, RCT	(low)
Haddad et al. (2015) [68]	226	48.9	65.6	13.0	TNFi	MDA	varying	Canada	Retrospective, single-center, cohort study	8
Queiro et al. (2018) [69]	50	50.4	46.0	8.2	UST	MDA	52	Spain	Retrospective, single-center, cohort study	6
Perrotta et al. (2016) [70]	75	52*	46.7	6.5*	ADA, ETA, GOL	MDA	52	Italy	Prospective, single-center, cohort study	9
Carvalho et al. (2017) [71]	180	52.3	45.6	11.9	ADA, ETA, IFX, GOL	EULAR response, DAS28	13, 26	Portugal	Retrospective, multicenter, cohort study	7
Vieira-Sousa et al. (2020) [72]	750	47.6	49.7	10.2	ADA, ETA, IFX, GOL	EULAR response	13, 26	Portugal	Prospective, multicenter, cohort study	8
Perrotta et al. (2020) [73]	70	45.7	55.7	7*	UST, SEC, IXE	MDA	26	Italy	Prospective, multicenter, cohort study	7
Eder et al. (2022) [74]	679	51.0	45.7	11.1	IXE	MDA, DAPSA, ACR	24, 52, 108, 156	International	Prospective, multicenter, RCT	(low)
Venerito et al. (2020) [75]	27	58.4	40.4	10.6	APT	DAPSA	26	Italy	Prospective, single-center, cohort study	7
Kavanaugh et al. (2014) [76]	504	50.1	47.9	7.7	APT	ACR20	16	International	Prospective, multicenter, RCT	(low)
Glintborg et al. (2011) [77]	764	47*	48.2	7*	ADA, ETA, IFX	EULAR response, ACR	26	Denmark	Prospective, multicenter, cohort study	7
Luchetti Gentiloni et al. (2023) [78]	126	56.5	31.7	7.7*	UPA	MDA	24	Italy	Prospective, multicenter, cohort study	9

^a^characteristics of PsA cohort within total study cohort, * = median, ^b^ since first symptoms (if specified), *NR* not reported, *NOS* Newcastle–Ottawa scale: scores ≥ 7–9, 4–6, < 4 are considered low, intermediate, and high risk in following statistics; *RoB 2* Risk of bias 2 tool, *TNFi* tumour necrosis factor inhibitor, *SEC* secukinumab, *ADA* adalimumab, *ABT* abatacept, *ETA* etanercept, *IFX* infliximab, *APT* apremilast, *GOL* golimumab, *CZP* certolizumab pegol, *UST* ustekinumab, IXE = ixekizumab, UPA = upadacitinib, csDMARD = conventional synthetic disease-modifying antirheumatic drug, *bDMARD* biological DMARD, *DAS28* Disease Activity Score 28, *CPDAI* Composite Psoriatic Disease Activity Index, *(c)DAPSA* clinical Disease Activity in Psoriatic Arthritis, *MDA* Minimal Disease Activity, *ACR* American College of Rheumatology, *EULAR* European Alliance of Associations of Rheumatology

### Risk of bias in included studies

The majority of the included studies had an observational cohort study design (81.1%). According to the NOS score, the risk of bias was low or moderate (6–9 points) for all observational studies. For all RCTs, except for Au et al. [46] with some concerns, the RoB 2 tool indicated a low risk of bias. The completed evaluation forms are available in Additional Tables S1-S2. The risk of bias assessments of the individual studies can be found in Table 1.

### Predictors of treatment response

The results of all meta-analyses performed, as well as data for the remaining predictors with fewer than four studies, are summarised in Table 2: Sex was analysed as a predictor in 18 of the 37 studies, CRP in 14, age in 13, and HAQ in 10 studies (Table 2). Furthermore, BMI, disease duration, DAPSA, DAS28, psoriasis, pretreatment, SJC, ESR, smoking, axial involvement, TJC, and PGA were explored in at least four studies (Table 2). Data for these 16 predictors originated from 27 of the 37 included studies.

**Table 2:** Predictors of treatment response

Predictor	Studies	*n*	OR (95% CI)	$\tau$^2^	I^2^	GRADE
*Demographics*						
Male sex	18 (7)	7′357	2.188 (1.912–2.503)	0.02	36.7%	low
Age	13 (5)	3′967	0.982 (0.975–0.99)	0	2.1%	low
BMI/obesity	9 (3)	4′688	0.98 (0.953–1.008)	0	0.0%	very low
Smoking	4 (5)	1′384	0.853 (0.672–1.082)	0	0.0%	very low
Alcohol, education	1	180	ns			
Year of prescription	1 (1)	750	+			
Age at PsA onset, country	(1)					
*Medical history*						
Disease duration	8 (9)	2′101	0.974 (0.95–1.0)	0.001	77.4%	very low
Pretreatment/treatment line	6	2′598	0.935 (0.389–2.249)	1.722	89.9%	very low
Fibromyalgia	2	358	-, -			
Metabolic syndrome + CVD	2 (3)	1′089	-, -			
Treatment duration	2	185	ns, +			
Other comorbidities	2 (3)	666	-, -			
Adverse effect as reason for previous TNFi stop	2	822	-, ns			
Thyreopathies	1	221	-			
IBD	1	75	ns			
FiRST	1	868	-			
Widespread non-arthritic pain	1	69	-			
Depression/anxiety	1	728	-			
Lack of effect as reason for previous TNFi stop	1 (1)	548	-			
Type of previous TNFi, concomitant infections	(1)					
*Disease activity/state*						
HAQ(-DI)	10 (6)	3′188	0.483 (0.336–0.696)	0.282	73.5%	very low
(c)DAPSA	7	2′082	0.789 (0.663–0.938)	0.07	71.9%	very low
DAS28	7 (3)	2′907	1.046 (0.796–1.374)	0.166	38.7%	very low
Psoriasis/PASI/BSA	7 (2)	3′525	0.898 (0.798–1.01)	0.006	30.0%	low
SJC	6 (5)	3′055	1.028 (0.859–1.229)	0.087	99.1%	very low
Axial involvement	4	1′014	0.335 (0.06–1.852)	2.996	73.3%	very low
TJC	4 (5)	3′008	0.97 (0.945–0.996)	0.000	11.6%	low
PGA	4 (5)	2′703	1.007 (0.998–1.016)	0.000	0.0%	low
Pain	3 (3)	1′759	+ , ns, ns			
Enthesitis	3 (4)	2′627	± , ns, ns			
Large joint involvement	2	511	-, -			
Dactylitis	2 (4)	1′292	± , +			
DJC	1	226	ns			
LEI	1 (1)	70	ns			
SDAI	1	135	ns			
Extraarticular manifestations	1 (1)	180	ns			
Uveitis	1	75	ns			
∆TSJ, ∆PEG	1	1′236	-			
BASFI	1	136	-			
PsAID-12	1 (2)	868	-			
FFbH	1	1′684	+			
PsA subtype	(5)					
Fatigue, BASDAI, PhGA	(2)					
DLQI, Ritchie Index	(1)					
*Serological*						
CRP	14 (7)	3′581	1.537 (1.111–2.125)	0.542	99.6%	very low
ESR	6 (3)	1′281	0.909 (0.725–1.14)	0.116	49.3%	very low
C3	1	55	-			
IFNγ, IL6, TNFα, IL17A, IL21	1	47	ns			
IL22	1	47	+			
IL23	1	47	ns			
Adiponectin, ENRAGE, IgA, IL16, Myeloperoxidase, PAP, VEGF	1	74	+			
Apolipoprotein CIII, IGF1, MMP3	1	74	ns			
Insulin, SGOT, Pyridinoline	1	74	-			
sCD40L	1	27	ns			
C4, FVII, Leptin	(1)					

Meta-analysis was performed for predictors with ≥ 4 studies. Studies who reported insignificant results without quantitative data were added in brackets. *N* number of patients, *OR* odds ratio, *CI* confidence interval, $\tau$^2^ tau squared, *I*^2^ Higgins & Thompson’s I^2^ statistic *ns* not significant, +  = positively correlated with predictor,—= negatively correlated with predictor; *BMI* body mass index, *PsA* psoriatic arthritis, *CVD* cardiovascular disease, *TNFi* tumour necrosis factor inhibitor, *IBD* inflammatory bowel disease, *FiRST* Fibromyalgia Rapid Screening Tool [79], *HAQ-DI* Health Assessment Questionnaire- Disability Index, *(c)DAPSA* (clinical) Disease Activity in Psoriatic Arthritis, *DAS28* Disease Activity Score 28, *PASI* Psoriasis Area and Severity Index, *BSA* Body Surface Area, *SJC* swollen joint count, *TJC* tender joint count, *PGA* Patient Global Assessment, *DJC* damaged joint count, *LEI* Leeds Enthesitis Index [80], *SDAI* Simplified Disease Activity Index for Rheumatoid Arthritis [81], ∆*TSJ* numeric differences between 28 tender and swollen joint count, ∆*PEG* numeric differences between patient’s and evaluator’s global assessment, *BASFI* patient’s and evaluator’s global assessment [82], *PsAID-12* Psoriatic Arthritis Impact of Disease 12-item questionnaire [83], *FFbH* Funktionsfragebogen Hannover [84], *BASDAI* Bath Ankylosing Spondylitis Disease Activity Index [85], *PhGA* Physician Global Assessment, *DLQI* Dermatology Life Quality Index [86], Ritchie Index [82], *CRP* C-reactive protein, *ESR* Erythrocyte sedimentation rate, *C3* complement component 3, *IFNγ* interferon gamma, *IL* interleukin, *TNFα* tumour necrosis factor alpha, *ENRAGE* S100 calcium-binding protein A12, *IgA* immunoglobulin A, *IGF-1* insulin-like growth factor 1, *PAP* prostatic acid phosphate, *SGOT* serum glutamic oxaloacetic transaminase, *VEGF* vascular endothelial growth factor, *MMP3* Matrix Metallopeptidase 3, *sCD40L* Soluble CD40 ligand, *C4* complement component 4, *FVII* coagulation factor VII

Predictors without data, which were only indicated as non-significant in the studies, are listed in brackets

**Fig. 2:**
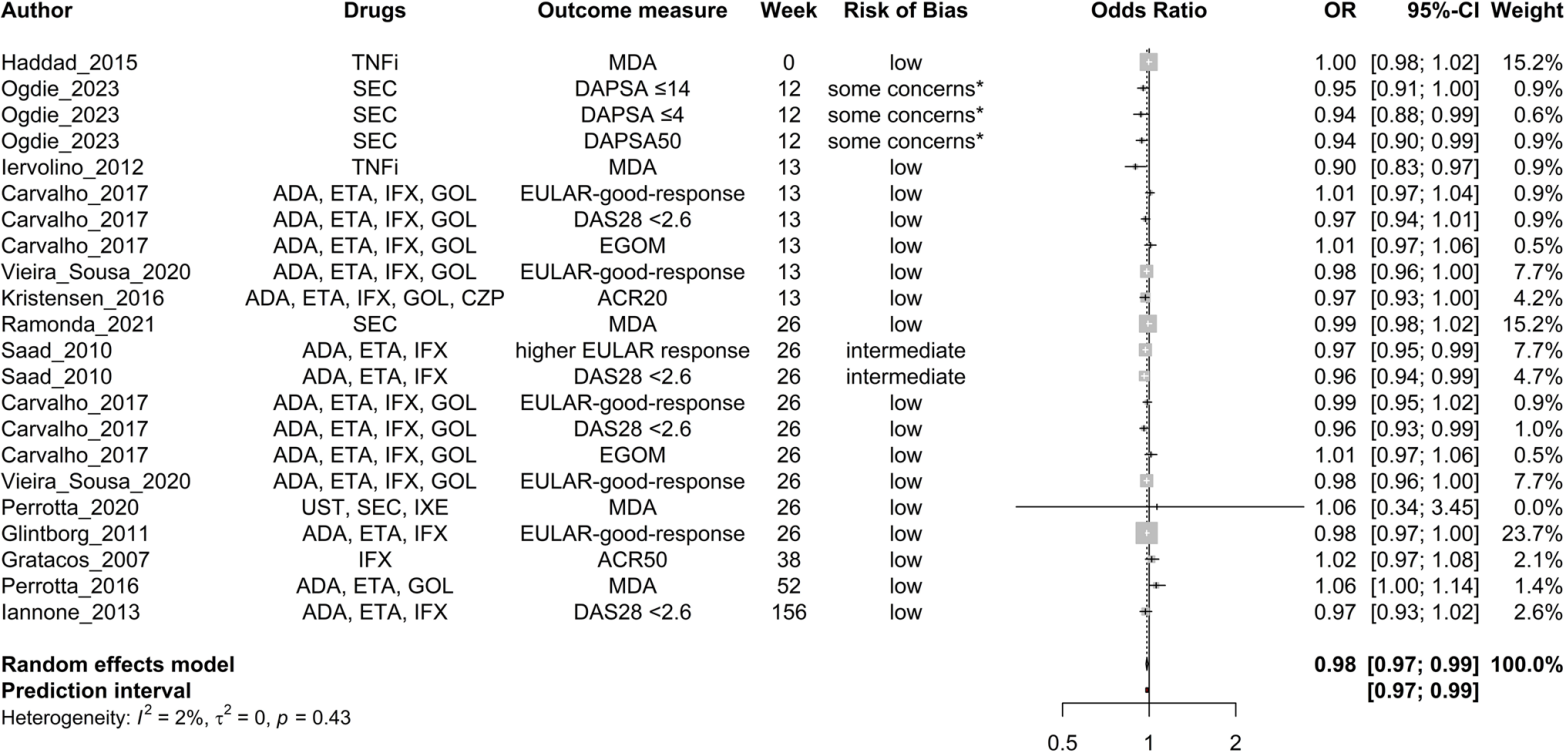
Forest Plot with Adjusted Weights and Pooled OR – Age. TNFi = tumour necrosis factor inhibitor, SEC = secukinumab, ADA = adalimumab, ETA = etanercept, IFX = infliximab, GOL = golimumab, CZP = certolizumab pegol, UST = ustekinumab, IXE = ixekizumab, MDA = minimal disease activity, DAPSA = Disease Activity in Psoriatic Arthritis, DAS28 = Disease Activity Score 28, EGOM = EULAR good/moderate response, ACR = American College of Rheumatology, EULAR = European Alliance of Associations of Rheumatology, OR = odds ratio, CI = confidence interval, I2 = Higgins & Thompson’s I2 statistic, t2 = tau squared

**Fig. 3:**
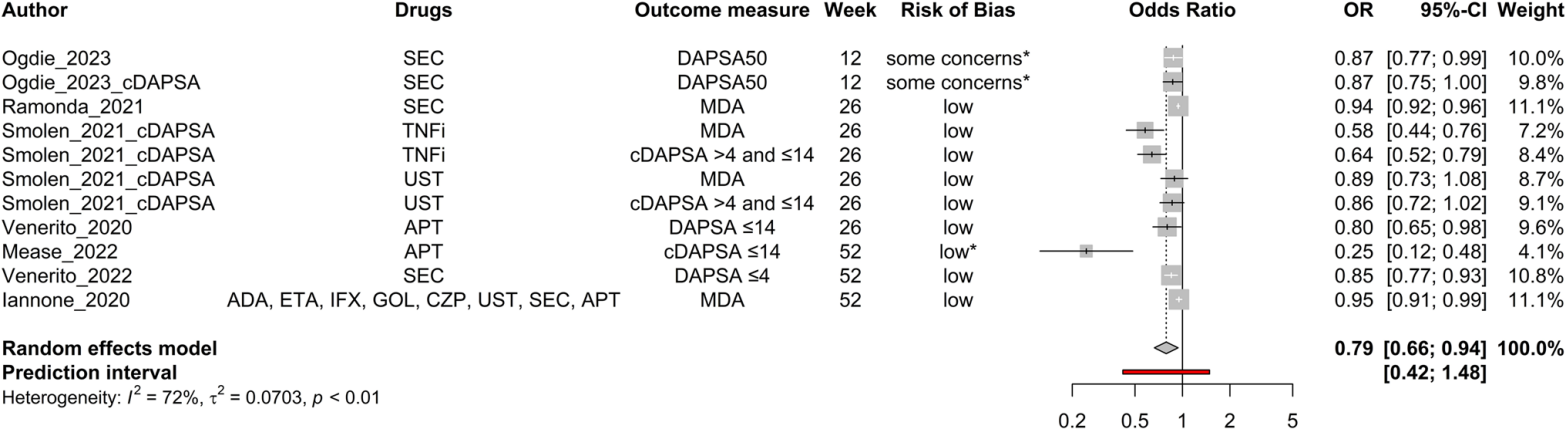
Forest Plot with Adjusted Weights and Pooled OR – DAPSA. SEC = secukinumab, TNFi = tumour necrosis factor inhibitor, UST = ustekinumab, APT = apremilast, ADA = adalimumab, ETA = etanercept, IFX = infliximab, GOL = golimumab, CZP = certolizumab pegol, (c)DAPSA = (Clinical) Disease Activity in Psoriatic Arthritis, MDA = minimal disease activity, OR = odds ratio, CI = confidence interval, I2 = Higgins & Thompson’s I2 statistic, t2 = tau squared

**Fig. 4:**
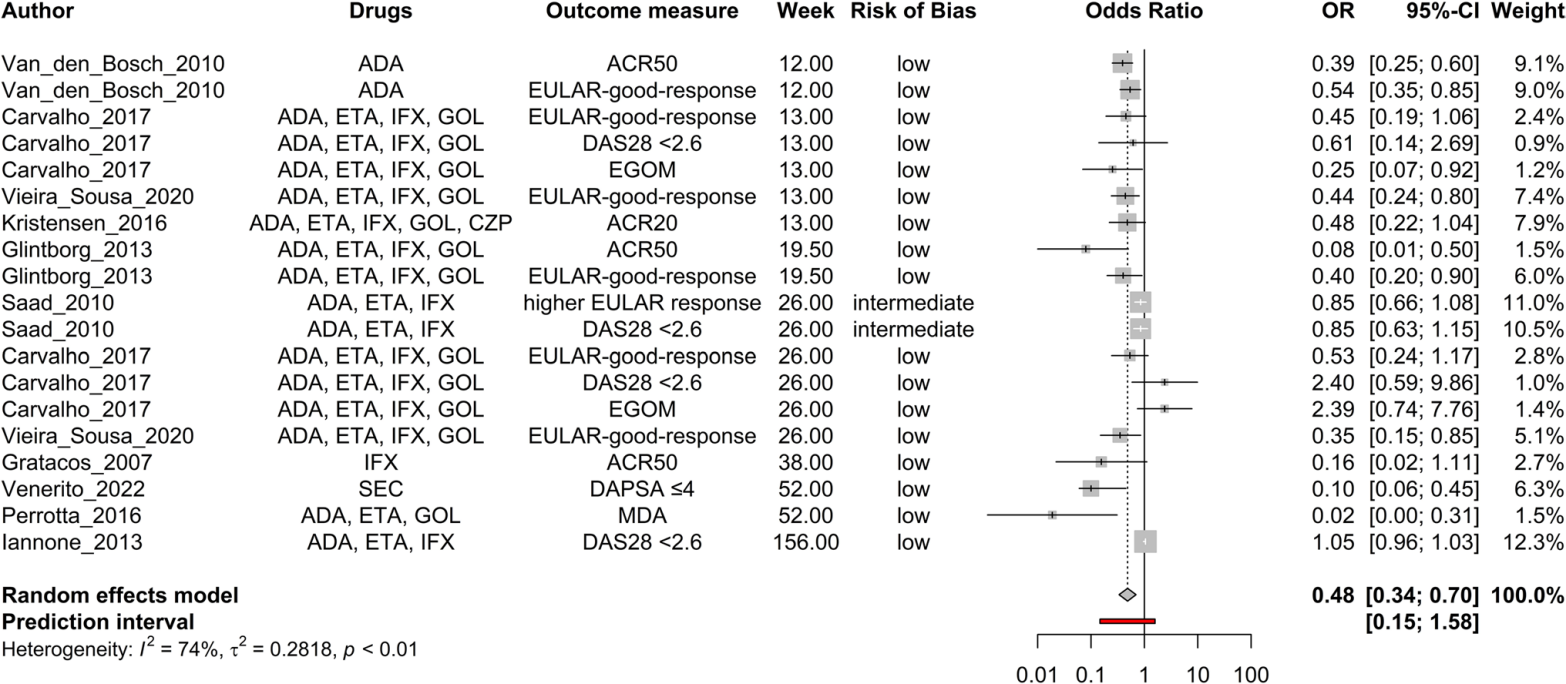
Forest Plot with Adjusted Weights and Pooled OR – HAQ. ADA = adalimumab, ETA = etanercept, IFX = infliximab, GOL = golimumab, CZP = certolizumab pegol, SEC = secukinumab, ACR = American College of Rheumatology, EULAR = European Alliance of Associations of Rheumatology, DAS28 = Disease Activity Score 28, EGOM = EULAR good/moderate response, DAPSA = Disease Activity in Psoriatic Arthritis, MDA = minimal disease activity, OR = odds ratio, CI = confidence interval, I2 = Higgins & Thompson’s I2 statistic, t2 = tau squared, HAQ = health assessment questionnaire

**Fig. 5:**
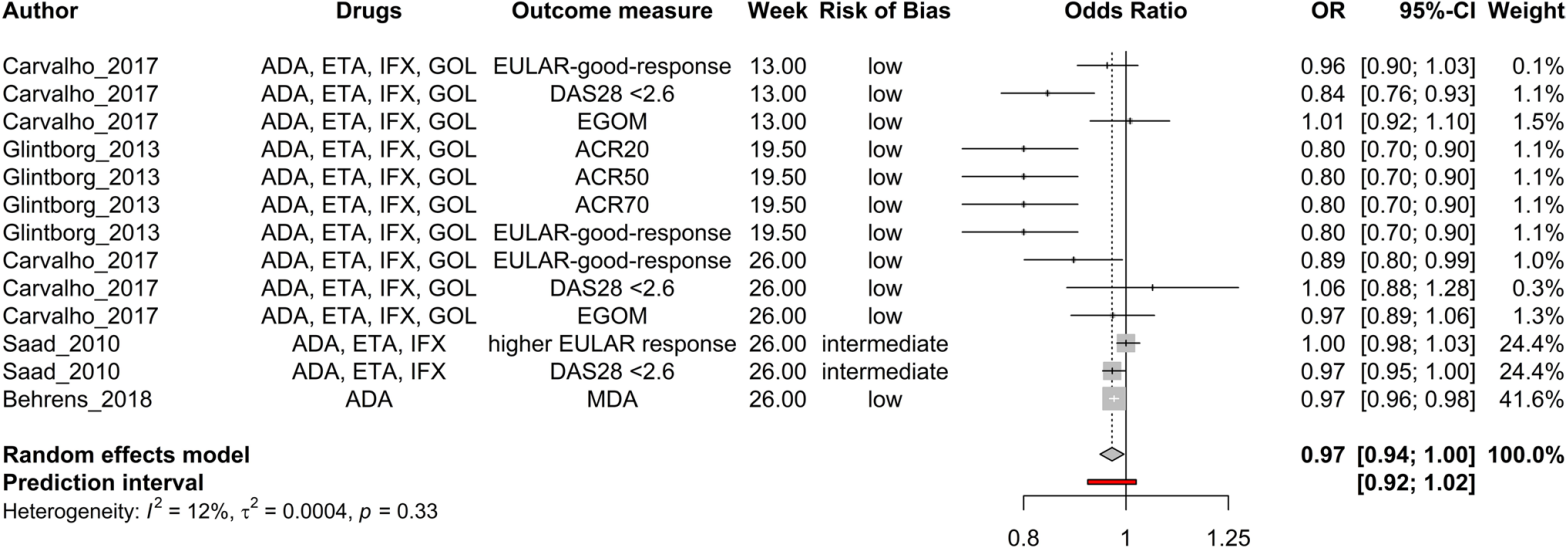
Forest Plot with Adjusted Weights and Pooled OR – TJC. ADA = adalimumab, ETA = etanercept, IFX = infliximab, GOL = golimumab, EULAR = European Alliance of Associations of Rheumatology, DAS28 = Disease Activity Score 28, EGOM = EULAR good/moderate response, ACR = American College of Rheumatology, MDA = minimal disease activity, OR = odds ratio, CI = confidence interval, I2 = Higgins & Thompson’s I2 statistic, t2 = tau squared, TJC = tender joint count

**Fig. 6:**
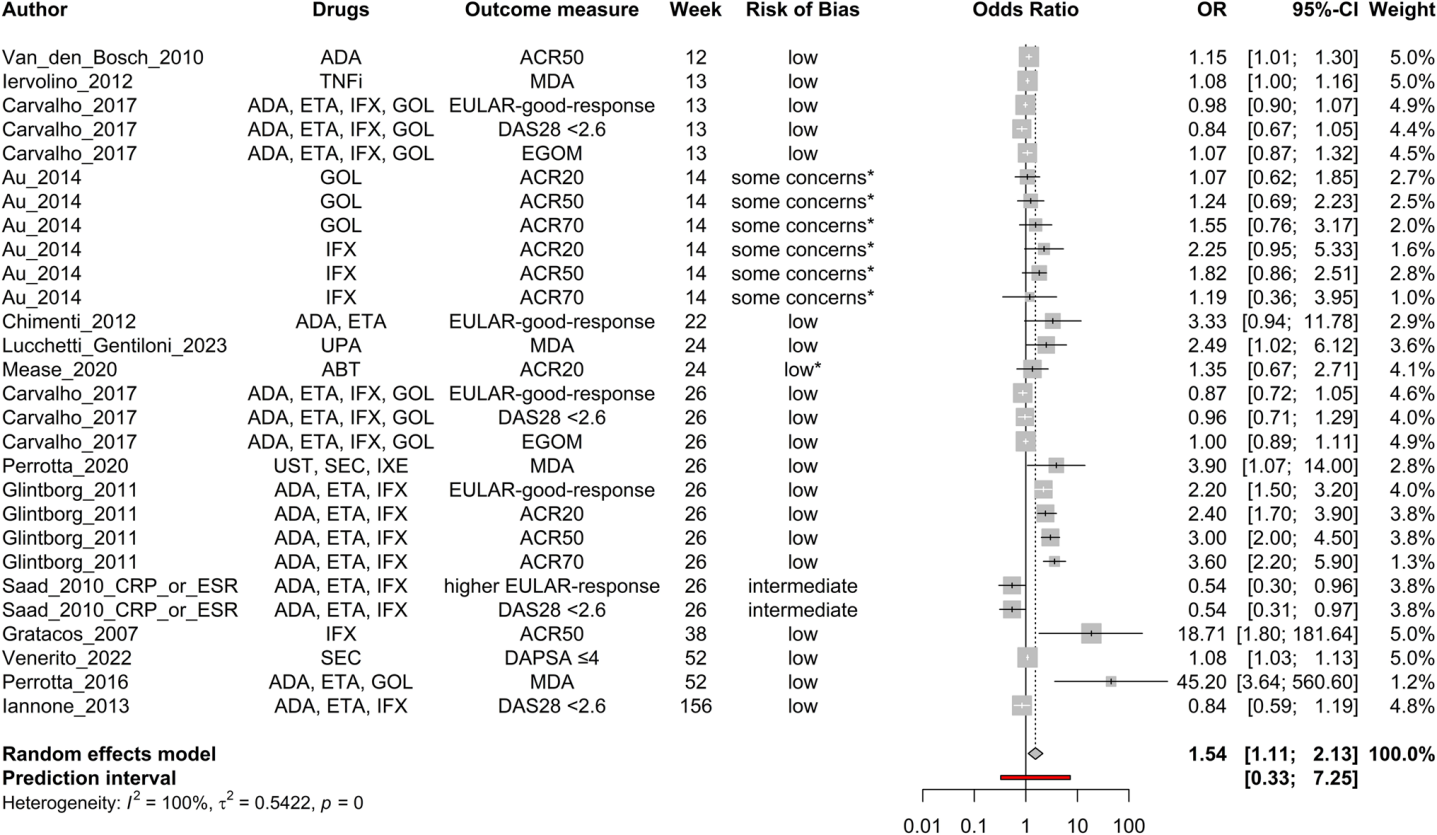
Forest Plot with Adjusted Weights and Pooled OR – CRP. ADA = adalimumab, TNFi = tumour necrosis factor inhibitor, ETA = etanercept, IFX = infliximab, GOL = golimumab, UPA = upatacitinib, ABT = abatacept, UST = Ustekinumab, SEC = secukinumab, IXE = ixekizumab, ACR = American College of Rheumatology, MDA = minimal disease activity, EULAR = European Alliance of Associations of Rheumatology, EGOM = EULAR good/moderate response, DAS28 = Disease Activity Score 28, DAPSA = Disease Activity in Psoriatic Arthritis, OR = odds ratio, CI = confidence interval, I2 = Higgins & Thompson’s I2 statistic, t2 = tau squared, C-reactive protein

**Fig. 7:**
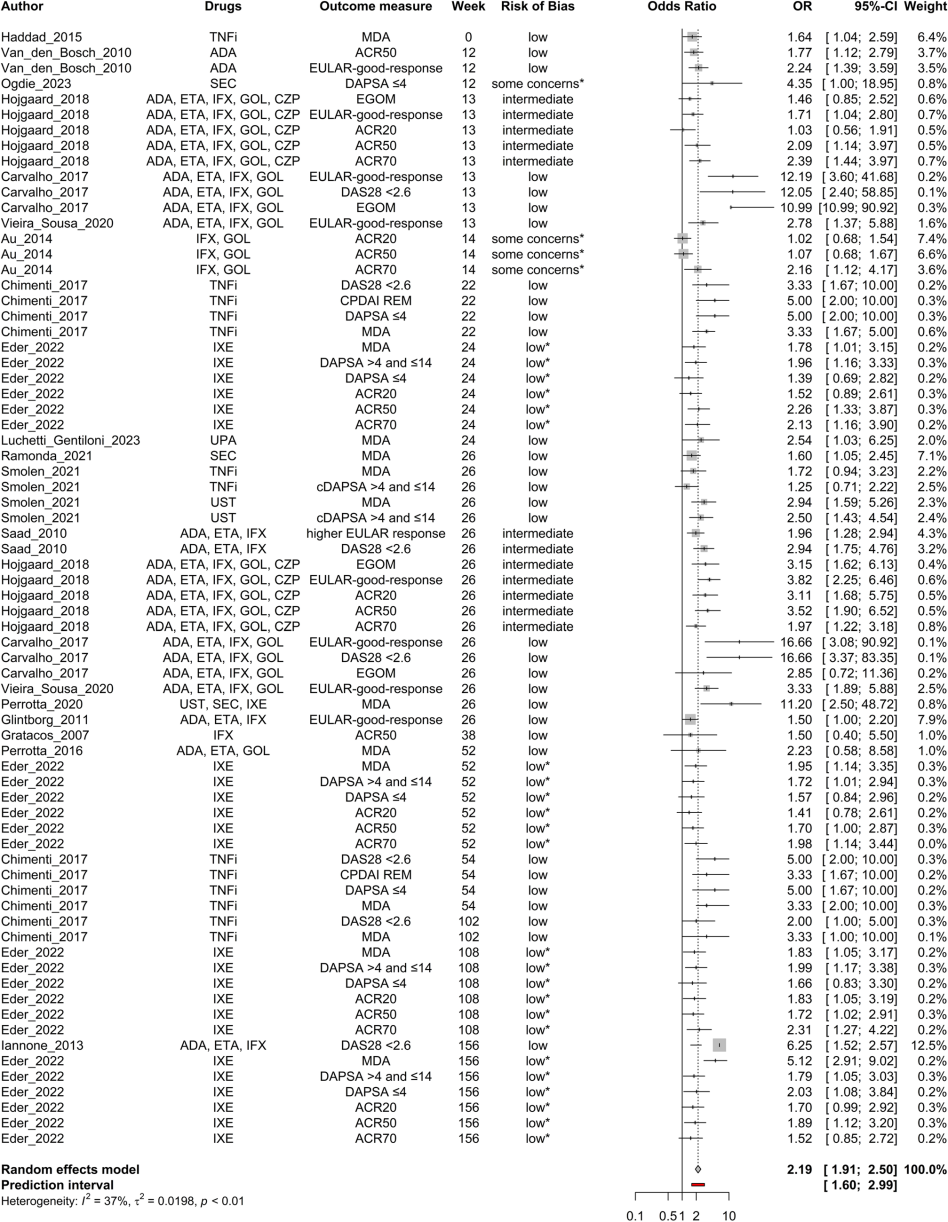
Forest Plot with Adjusted Weights and Pooled OR – Male Sex. TNFi = tumour necrosis factor inhibitor, ADA = adalimumab, SEC = secukinumab, ETA = etanercept, IFX = infliximab, GOL = golimumab, IXE = ixekizumab, UPA = upadacitinab, UST = ustekinumab, CZP = certolizumab pegol, MDA = minimal disease activity, ACR = American College of Rheumatology, EULAR = European Alliance of Associations of Rheumatology, (c)DAPSA = (Clinical) Disease Activity in Psoriatic Arthritis, EGOM = EULAR good/moderate response, DAS28 = Disease Activity Score 28, CPDAI REM = Composite Psoriatic Disease Activity Index remission, OR = odds ratio, CI = confidence interval, I2 = Higgins & Thompson’s I2 statistic, t2 = tau squared

Age (Fig. 2), baseline DAPSA score (Fig. 3), baseline HAQ score (Fig. 4), and baseline TJC score (Fig. 5) were found to be negatively correlated with treatment response. Baseline CRP (Fig. 6) and male sex (Fig. 7) were identified as positive predictors. The ORs for BMI, smoking, disease duration, pretreatment, baseline DAS28, skin disease, baseline SJC, axial disease, baseline PGA, and baseline ESR were not statistically significant (Additional Figures S2A-T). The forest plots below show the results of the quantitative analyses with adjusted weights and pooled effect sizes (Figs. 2–7). Visually, no correlation between predictor strength and the time point of the outcome measurement was observed for any predictor. The forest plots with qualitative analysis based on original weights and without pooled effect sizes, as well as the forest plots of the predictors with statistically nonsignificant results, are included in Additional Figures S1–S2.

For predictors with fewer than four studies, no quantitative meta-analyses were performed. Nevertheless, certain trends can be deduced from the results of individual studies. For statistically significant results, positive or negative predictions are listed in Table 2. The consistent results from two independent studies for each of these predictors indicate that concomitant fibromyalgia, comorbid metabolic syndrome or other CVD, higher overall comorbidity, and involvement of large joints are associated with a tendency toward poorer treatment response.

Heterogeneity was low for age (Fig. 2), BMI, smoking status, TJC (Fig. 5), and PGA. The DAS28, sex (Fig. 7), ESR, psoriasis, axial disease, disease duration, treatment line, HAQ (Fig. 4), DAPSA (Fig. 3), SJC, and CRP (Fig. 6) were moderate.

Outliers were identified for age, BMI, CRP, DAPSA, DAS28, ESR, HAQ, PGA, psoriasis, sex, SJC, TJC, and treatment line. The adjusted ORs, following the exclusion of the identified outliers, are summarised in Table 3. The ORs of age (∆OR ± 0), BMI (+ 0.006), psoriasis (-0.005), and TJC (+ 0.006) remained essentially unchanged. The strength of DAPSA (+ 0.077), ESR (+ 0.053), and HAQ (+ 0.019) became slightly weaker. The predictive values observed for CRP (+ 0.288), sex (+ 0.349), the SJC (+ 0.026), and the treatment line (-0.159) were even slightly more pronounced after adjustment. Only for DAS28 (-0.057) did the trend change from a positive to a negative association, but the difference was still not statistically significant.

**Table 3:** Exclusion of outliers

Predictor	Unadjusted OR	Outlier(s)	OR after exclusion of outliers (95% CI)	$\tau$^2^	I^2^
Age	0.982	Perrotta et al. (2016) [70] Iervolino et al. (2012) [58]	0.982 (0.974–0.99)	0	0.0%
BMI	0.98	Venerito et al. (2022) [45] Hojgaard et al. (2016) [55] Vieira-Sousa et al. (2020) [72]	0.986 (0.959–1.015)	0	0.0%
CRP	1.537	Perrotta et al. (2016) [70] Carvalho et al. (2017) [71] Glintborg et al. (2011) [77] Saad et al. (2010) [63]	1.825 (1.279–2.606)	0.443	99.7%
DAPSA	0.789	Mease et al. (2022) [48]	0.866 (0.803–0.933)	0.007	56.7%
DAS28	1.046	Glintborg et al. (2013) [50] Carvalho et al. (2017) [71]	0.989 (0.967–1.012)	0	0.0%
ESR	0.909	Perrotta et al. (2016) [70]	0.962 (0.888–1.042)	0.011	34.3%
HAQ	0.483	Iannone et al. (2013) [49] Perrotta et al. (2016) [70] Gratacos et al. (2007) [57]	0.502 (0.369–0.683)	0.112	43.5%
Psoriasis	0.898	Smolen et al. (2021) [62]	0.893 (0.792–1.008)	0.007	32.9%
Sex	2.188	Hojgaard et al. (2018) [64] Carvalho et al. (2017) [71] Eder et al. (2022) [74] Au et al. (2014) [46]	2.537 (2.218–2.901)	0.006	17.4%
SJC	1.028	Ogdie et al. (2023) [66]	1.054 (0.892–1.246)	0.072	99.2%
TJC	0.97	Carvalho et al. (2017) [71] Glintborg et al. (2013) [50]	0.976 (0.967–0.985)	0	0.0%
Treatment line	0.935	Luchetti Gentiloni et al. (2023) [78]	0.776 (0.315–1.908)	1.599	86.8%

*OR* odds ratio, $\tau$^2^ tau squared, *I*^2^ Higgins & Thompson’s I^2^ statistic, *BMI* body mass index, *CRP* C-reactive protein, *DAPSA* Disease Activity in Psoriatic Arthritis, *DAS28* Disease Activity Score 28, *ESR* erythrocyte sedimentation rate, *HAQ* Health Assessment Questionnaire, *SJC* swollen joint count, *TJC* tender joint count

Influential data points were identified via the leave‒one-out method (Table 4). Apart from two exceptions, the direction and estimated magnitude of the predictive values remained unchanged. Leaving out Gratacos et al. [57] weakened the positive predictive power of CRP to such an extent that the result was no longer statistically significant (CI 95% 1.00–1.52). Psoriasis, on the other hand, achieved previously non-existent statistical significance as a negative predictor when Behrens et al. [54] was excluded.

**Table 4:** Influential data points

Predictor	Unadjusted OR	Influential data point	OR after leave-one-out method (95% CI)	I^2^
CRP	1.537	Gratacos et al. (2007) [57]	1.23 (1.00–1.52)	43%
DAPSA	0.789	Mease et al. (2022) [48]	0.866 (0.803–0.933)	56.7%
Disease duration	0.974	Haddad et al. (2015) [68]	1.00 (0.98–1.01)	0%
ESR	0.909	Haddad et al. (2015) [68]	0.97 (0.86–1.10)	33%
ESR	0.909	Perrotta et al. (2016) [70]	0.96 (0.89–1.04)	34%
HAQ	0.483	Venerito et al. (2022) [45]	0.60 (0.42–0.87)	64%
HAQ	0.483	Iannone et al. (2013) [49]	0.48 (0.30–0.75)	55%
Psoriasis	0.898	Behrens et al. (2018) [54]	0.79 (0.68–0.92)	0%
Psoriasis	0.898	Venerito et al. (2022) [45]	0.99 (0.97–1.00)	0%
SJC	1.028	Van den Bosch et al. (2010) [61]	1.00 (0.92–1.08)	27%
Smoking	0.853	Ramonda et al. (2021) [60]	0.78 (0.59–1.02)	0%
Smoking	0.853	Saad et al. (2010) [63]	0.79 (0.60–1.04)	0%
Sex	2.188	Iannone et al. (2013) [49]	1.81 (1.59–2.06)	0%
TJC	0.97	Glintborg et al. (2013) [50]	0.97 (0.97–0.98)	2%
TJC	0.97	Saad et al. (2010) [63]	0.97 (0.95–0.99)	3%

*OR* odds ratio, *CI* confidence interval, *I*^2^ Higgins & Thompson’s I^2^ statistic, *CRP* C-reactive protein, *DAPSA* Disease Activity in Psoriatic Arthritis, *ESR* erythrocyte sedimentation rate, *HAQ* Health Assessment Questionnaire, *SJC* swollen joint count, *TJC* tender joint count

Subgroup analyses revealed statistically significant between-subgroup differences for CRP (outcome measure, P = 0.004), DAPSA (drug, 0.003), HAQ (outcome measure, < 0.0001; drug, 0.001), psoriasis (outcome measure, 0.006; drug, 0.018), sex (outcome measure, < 0.0001), SJC (drug, 0.014), TJC (outcome measure, 0.036), and treatment line (outcome measure, 0.024). For analyses with statistically significant differences, details of the individual subgroups can be found in Table 5.

**Table 5:** Subgroup analyses based on outcome measures and drugs

Predictor	Subgroup	Number of data points	OR (95% CI)	$\tau$^2^	I^2^	*P*-value for between-subgroup difference
Age	*Outcome measure*					*0.463*
	*Drug*					*0.832*
BMI	*Outcome measure*					*0.169*
	*Drug*					*0.124*
CRP	*Outcome measure*					*0.004*
	DAPSA	1	1.08 (1.001–1.166)	-	-	
	DAS28	4	0.816 (0.629–1.058)	0	0.0%	
	EULAR	7	1.079 (0.764–1.524)	0.134	41.5%	
	MDA	4	3.336 (0.842–13.223)	1.554	80.7%	
	ACR	12	2.363 (1.378–4.054)	0.534	99.4%	
	*Drug*					*0.925*
DAPSA	*Outcome measure*					*0.32*
	*Drug*					*0.003*
	TNFi	2	0.464 (0.146–1.472)	0.632	90.6%	
	ILi	6	0.925 (0.897–0.954)	0.000	14.1%	
	other	1	0.95 (0.912–0.99)	-	-	
	mixed	2	0.617 (0.488–0.781)	0	0.0%	
DAS28	*Outcome measure*					*0.086*
	*Drug*					*0.302*
Disease duration	*Outcome measure*					*0.412*
	*Drug*					*0.925*
ESR	*Outcome measure*					*0.964*
	*Drug*					*-*
HAQ	*Outcome measure*					< *0.0001*
	DAPSA	1	0.1 (0.037–0.274)	-	-	
	DAS28	4	1.049 (1.013–1.086)	0	0.0%	
	ACR	4	0.381 (0.24–0.606)	0	0.0%	
	EULAR	9	0.663 (0.509–0.862)	0	0.0%	
	MDA	1	0.019 (0.001–0.308)	-	-	
	*Drug*					*0.001*
	TNFi	18	0.587 (0.437–0.788)	0.128	64.1%	
	ILi	1	0.1 (0.037–0.274)	-	-	
Psoriasis	*Outcome measure*					*0.006*
	DAPSA	5	0.62 (0.458–0.839)	0	0.0%	
	MDA	9	0.961 (0.88–1.049)	0.003	12.2%	
	*Drug*					*0.018*
	TNFi	8	0.798 (0.667–0.955)	0.005	9.5%	
	ILi	5	0.986 (0.973–1.0)	0	0.0%	
	other	1	0.462 (0.188–1.134)	-	-	
Sex	*Outcome measure*					< *0.0001*
	DAS28	7	5.663 (4.446–7.215)	0	0.0%	
	CPDAI	2	4.17 (0.627–27.717)	0	0.0%	
	DAPSA	13	2.049 (1.294–3.243)	0	0.0%	
	MDA	14	1.959 (1.53–2.507)	0	0.0%	
	ACR	23	1.371 (1.093–1.719)	0	0.0%	
	EULAR	13	2.027 (1.576–2.607)	0	0.0%	
	*Drug*					*0.924*
SJC	*Outcome measure*					*0.094*
	*Drug*					*0.014*
	TNFi	11	1.077 (0.919–1.262)	0.062	99.3%	
	ILi	2	0.417 (0.199–0.872)	0	0.0%	
Smoking	*Outcome measure*					*0.406*
	*Drug*					*0.19*
TJC	*Outcome measure*					*0.036*
	EULAR	6	0.994 (0.961–1.028)	0	0.0%	
	DAS28	3	0.968 (0.935–1.002)	0	0.0%	
	ACR	3	0.800 (0.692–0.925)	0	0.0%	
	MDA	1	0.974 (0.965–0.984)	-	-	
	*Drug*					*-*
Treatment line	*Outcome measure*					*0.024*
	MDA	6	0.827 (0.167–4.091)	3.459	93.4%	
	EULAR	1	0.559 (0.338–0.925)	-	-	
	DAPSA	2	1.238 (0.614–1.498)	0.174	68.0%	
	ACR	1	2.075 (1.019–4.227)	-	-	
	*Drug*					*0.056*

*OR* odds ratio, *CI* confidence interval, $\tau$^2^ tau squared, *I*^2^ Higgins & Thompson’s I^2^ statistic, *BMI* body mass index, *CRP* C-reactive protein, *DAPSA* Disease Activity in Psoriatic Arthritis, *DAS28* Disease Activity Score 28, *ESR* erythrocyte sedimentation rate, *HAQ* Health Assessment Questionnaire, *SJC* swollen joint count; *TJC* tender joint count, *EULAR* European Alliance of Associations for Rheumatology, *MDA* minimal disease activity, *ACR* American College of Rheumatology, *CPDAI* Composite Psoriatic Disease Activity Index, *TNFi* tumour necrosis factor inhibitor, *ILi* interleukin inhibitor

### Reporting biases

No evidence for publication bias was found for age, axial disease, DAS28, disease duration, ESR, PGA, psoriasis, smoking, treatment line, or SJC. Some evidence was found for BMI, CRP, HAQ, and sex. Publication bias was strongly suspected for DAPSA and TJC (Table 6).

**Table 6:** Publication bias

Predictor	Funnel plot: visual asymmetry	K	t-value	${P}_{t}$	z-value	${P}_{z}$
Age	No		-0.33	0.75	-0.17	0.86
Axial disease	No	< 10				
DAS28	No		0.61	0.55	1.65	0.10
Disease duration	No		0.99	0.34	-0.89	0.37
ESR	No		-1.07	0.31	-0.07	0.95
PGA	No	< 10				
Psoriasis	No		-1.3	0.22	0.49	0.62
Smoking	No		-0.27	0.79	-0.06	0.95
Treatment line	No		0.08	0.94	0	1.00
SJC	No		0.17	0.87	9.43	0.67
BMI	Yes		-4	0.00	-0.62	0.54
CRP	Yes		-1.34	0.19	4.35	0.00
HAQ	No		-2.09	0.03	-0.24	0.81
Sex	Yes		1.77	0.08	4.47	0.00
DAPSA	Yes		-4.82	0.00	-2.57	0.01
TJC	Yes		-2.58	0.03	-2.34	0.02

*K* data points, *t-value* Egger’s regression test, *P* p-value, *z-value* Begg and Mazumdar test, *DAS28* Disease Activity Score 28, *ESR* erythrocyte sedimentation rate, *PGA* patient global assessment, *SJC* swollen joint count, *BMI* body mass index, *CRP* C-reactive protein, *HAQ* Health Assessment Questionnaire, *DAPSA* Disease Activity in Psoriatic Arthritis, *TJC* tender joint count

Funnel plots for all the predictors are depicted in Additional Figures S3A-P. ORs adjusted for funnel plot asymmetry are depicted in Table 7. For BMI, sex, and TJC, the adjusted ORs were almost identical to the unadjusted ORs. For the DAPSA and HAQ, the adjusted ORs were closer to 1 than the unadjusted ORs were and were no longer statistically significant. The OR after the Trim and Fill method for CRP was substantially greater than the unadjusted OR (Table 7).

**Table 7:** Adjusted OR by using Duval % Tweedie trim and fill method

Predictor	Unadjusted OR	Adjusted OR	95% CI of adjusted OR	Adjusted $\tau$^2^	Adjusted I^2^
BMI	0.98	0.982	0.955–1.01	0	0.0%
CRP	1.537	10.916	4.92–24.218	6.791	99.9%
DAPSA	0.789	0.929	0.749–1.152	0.183	77.6%
HAQ	0.483	0.892	0.495–1.606	1.703	78.4%
Sex	2.188	2.187	1.895–2.524	0.273	68.0%
TJC	0.97	0.972	0.917–1.03	0.006	31.1%

*OR* odds ratio, *CI* confidence interval, $\tau$^2^ tau squared, *I*^2^ Higgins & Thompson’s I^2^ statistic, *BMI* body mass index, *CRP* C-reactive protein, *DAPSA* Disease Activity in Psoriatic Arthritis, *HAQ* Health Assessment Questionnaire, *TJC* tender joint count

### Certainty of evidence

According to the GRADE assessment and the observational nature of the studies, the overall CoE for all the predictors was low or very low (Table 2). The assessment of the individual variables of GRADE can be found in Additional Table S3.

## Discussion

This systematic review and meta-analysis aimed to identify predictors of treatment response to biological and targeted synthetic disease-modifying antirheumatic drugs in PsA patients, with a view toward future AI tool development. Several baseline factors significantly influenced treatment response: older age, baseline DAPSA, HAQ, and TJC scores were linked to poorer outcomes, while male sex and higher baseline CRP improved response. However, the effect sizes for age, DAPSA, and TJC were small, raising questions about their clinical relevance. Factors such as overweight, smoking, longer disease duration, advanced treatment line, skin and axial involvement, and higher ESR showed trends toward negative outcomes but lacked statistical significance. Higher DAS28, SJC, and PGA scores were positively associated with response, though not statistically significant. Table 8 summarizes significant and borderline predictors for further analysis. No effect was observed regarding the timing of response measurement or therapy duration for any predictor. The summarised patient characteristics align with previously published numbers of PsA cases and are considered to be equally common among men and women [87]. A total of 46.3% of the patients in the included studies were male. Karmacharya et al. et al. [88] reported a mean age of 46.4 years for incident cases of PsA. It is known that PsA often starts between 30 and 50 years of age [89]. The calculated median age in the included studies was 48.0 years for this review.

**Table 8:** Predictors of better treatment response

Statistically significant	Potential
Younger age	BMI
Higher baseline CRP	Disease duration
Lower baseline DAPSA	Skin affection
Lower baseline HAQ	Smoking
Male sex	
Lower baseline TJC	

*CRP* C-reactive protein, *DAPSA* Disease Activity in Psoriatic Arthritis, *HAQ* Health Assessment Questionnaire, *TJC* tender joint count, *BMI* body mass index

Because the results remained mostly consistent across different sensitivity analyses, they can be considered robust overall. Nevertheless, associations of certain predictors, including CRP, which exhibited a strong dependence on the study of Gratacos et al. [57], as well as DAPSA and HAQ, were no longer statistically significant after correction for publication bias and therefore need to be interpreted with caution. The substantial heterogeneity of the data should also be taken into account because it weakens the generalizability of the pooled effect size due to the loss of information from studies with substantially different results [39]. For several predictors or their subgroups, the number of data points was small. This should be considered for interpretation, especially for subgroup analyses. Furthermore, the varying components of the different composite scores should be considered. For example, the treatment response of patients with high HAQ scores could have been lower when the ACR score was used than when the DAS28 and EULAR scores were used, as the HAQ is only part of the calculations for the ACR score and not for the other two scores [30].

Despite the influential case of Gratacos et al. [57], the data, even more so after adjustments for outliers or publication bias, support CRP as a positive predictor of treatment response. This result aligns with earlier reviews by Magee et al. [90] and Mekhail et al. [91]. Houttekiet et al. [92] underscored the presence of conflicting findings, partly attributed to the routine use of minimal CRP levels as enrollment criteria in clinical trials. Consequently, while CRP may influence future treatment decisions, further validation with real-world data is required. Patients with elevated baseline CRP levels may exhibit a greater inflammatory burden, suggesting more active disease and potentially a more pronounced response to anti-inflammatory therapies. In the review of Magee et al. [90], only studies on TNFis reported evidence that CRP is a predictor of treatment response, and studies by Siebert et al. [93] on IL12/23 did not. The subgroup analysis did not confirm any significant differences between different drug categories (see Table 5). No clear pattern was recognizable among the outcome measures with or without integrated CRP. Thus, the reasons for the significant subgroup analysis based on the outcome measure used (see Table 5) remain unclear.

The results for the ESR indicate the opposite, i.e., rather, suggest that normal values are a predictor of treatment response. Overall, the ESR has been studied far less as a predictor than the CRP (see Table 2). One possible explanation for the inverse correlation could be the naturally higher ESR values in women [94], who generally show a lower treatment response than men do (see below).

In addition to CRP, Mekhail et al. [91] described younger age, male sex, a lower HAQ score and the absence of obesity as positive predictors of treatment response but did not perform a meta-analysis due to high data heterogeneity. These statements are largely consistent with the results of this review (see Table 2). Sex differences will be discussed in the following paragraph. The superior treatment response of patients with preserved functionality, measured as a lower HAQ score, illustrates that not all disabilities caused by PsA are reversible. Assuming that the onset of the disease occurs at a similar age for all patients, older age increases the likelihood of a longer disease duration, irreversible damage, reduced functionality, and comorbidities [2]. Disease duration and the number of comorbidities tended to be negative predictors, but disease duration did not reach statistical significance, and comorbidities were analysed in fewer than four studies and therefore not evaluated quantitatively. Obesity and metabolic syndrome are common comorbidities of PsA [2, 95]. While a poorer response to TNFis has been shown in obese patients with RA or psoriasis, the corresponding data for PsA are still unclear [95]. Both BMI and metabolic comorbidities tended to be negative predictors but did not reach statistical significance, or a sufficient number of studies were included in the quantitative meta-analysis.

The poorer treatment response of women than men is known for PsA [11, 96] and was also evident in this review. The reasons for these sex-related differences are still the subject of research [71]. To date, there are only hypotheses [71] for shorter drug survival and an increased incidence of adverse events among women [35]. A few of them are addressed here: Earlier studies have shown a delayed start of bDMARDs for women [97]. Psychophysiological factors, such as central sensitisation or the perception of pain and fatigue, may influence the assessment of TJC and PRO, thus weakening the treatment response [96, 98]. Hojgaard et al. [64] reported that the associations between sex and outcome were independent of known risk factors such as PRO, disease activity, comorbidities, and lifestyle. This finding suggests the presence of biological factors such as genetic or hormonal differences [96]. According to Ortolan et al. [51], some authors suggest that women treated with SEC are less inferior than those treated with TNFis are, but the corresponding subgroup analysis revealed no significant differences (Table 5). The subgroup differences based on the outcome measure remain without a recognizable pattern and therefore without a clear explanation (Table 5).

Evidence regarding the influence of smoking on the development and progression of disease, as well as treatment response, is scarce and conflicting [47]. The four studies included in the analysis pointed in the direction of a poorer response of smokers, but the difference was not statistically significant.

Patients with comorbid fibromyalgia show greater perceptions of pain and fatigue and therefore increased self-reported assessments of disease activity, such as the HAQ [99]. “Almost no PsA patients with comorbid fibromyalgia achieve clinical disease remission upon biologic therapy” [99]. Unfortunately, the literature search did not yield enough studies for a meta-analysis, but the studies on fibromyalgia and widespread nonarthritic pain [45, 99, 100] confirmed these statements with statistical significance (Table 2). In a complementary manner to the previous section, the higher prevalence of fibromyalgia and generalised pain syndrome among women with PsA should be mentioned as another factor possibly contributing to the sex differences [71].

In addition to sex, Ogdie et al. [96] described baseline disease activity as the predictor with the most consistent data. In this context, DAPSA, DAS28, TJC, and SJC were analysed. Higher DAPSA and TJC values predict a poorer response to therapy. It seems understandable that a higher disease burden with the same therapy is less likely to achieve low activity or even remission. The DAS28 and SJC trended in the other direction but were not statistically significant.

Skin involvement and psoriasis tended toward a negative correlation, but the difference was not statistically significant. The negative predictor strength was more pronounced in the subgroup analysis for TNFis than for ILis (Table 5), which suggests that skin involvement is a greater problem under TNFis. This finding is consistent with the assumption that the IL-17 signature is stronger in the skin than in the joints and that SEC is therefore a good choice for patients with pronounced skin disease [17, 51]. The results of the subgroup analysis by outcome measure were expected to reveal a greater effect size for MDA, as the BSA or PASI are included directly in the MDA but not in the DAPSA [30]. An indirect explanation for the inverse result could be the strong influence of the skin condition on the PGA [101]. The PGA is included in both scores, but only 5 out of 7 requirements need to be met to achieve the MDA so that this treatment goal can also be achieved without successful improvement of the skin and PGA.

Because of individual disease manifestations, more subtype-specific data with appropriate outcome measures should be collected. Iannone et al. [31], for example, reported that axial disease represents a distinct subtype. The results displayed in Table 2 indicate a reduced response to therapy with axial involvement, but the difference was not statistically significant.

Biomarkers, with their objectivity, quantifiability, accuracy and reproducibility, are perfect predictors [13]. However, there are no validated predictive biomarkers in PsA yet [102]. Previous studies have shown that, most likely, no single parameter but panels of predictors will be used for the choice of treatment in the future [10]. In the proceedings of the 2017 Group of Research and Assessment of Psoriasis and Psoriatic Arthritis (GRAPPA), the Collaborative Research Network Meeting GRAPPA defined enabling personalised and stratified therapeutic approaches through the identification of predictors of treatment response for PsA and psoriasis as one of four research priorities [103]. It is clear that “[…] the treatment of specific pathways and, thereby, distinct patient subgroups, i.e., precision medicine, should be favoured over the treatment of mere indications” [22].

Although ML has already found its way into the healthcare system in other settings, it is still challenging to use it to predict the development, progression or treatment response in PsA patients [104]. Applications for verifying suspected predictors [45], incorporating identified predictors into clinical tools or even for discovering new potential predictors are imaginable [105]. Unsupervised AI tools without predefined predictors such as "PredictAI" have already shown great promise for detecting undiagnosed PsA and thus shortening treatment delay [106].

In the following, the focus will be on the development of tools for clinical use. To date, research, including our review to some extent, has assumed direct and isolated associations between predictors and outcomes, but ML is promising for the implementation of complex, multiparametric, nonlinear, and multidimensional decision algorithms [45]. Supervised learning algorithms have “[…] been widely tested for predicting treatment outcomes [27]. For predictive models, tree-based algorithms seem to be a good choice [45]. The end product of the data obtained in this review could be imagined in such a way that the raw patient data containing predictors such as age, sex and baseline disease activity are provided by the treating physician and that the algorithm delivers probability scores for the patient’s response to individual medications.

Future research should prioritise well-designed prospective studies with desirably standardised outcome measures to strengthen the robustness of the identified predictive factors. In addition, further potential predictors, especially genetic and molecular biomarkers, should be investigated to create enough homogeneous data for further meta-analyses, since PsA has a strong genetic component [8]. Increasing amounts of specific data would also help to obtain more reliable results from subgroup analyses for individual drug categories. Treatment monitoring to identify early clinical outcome measures as predictors of long-term response and support in the decision on the continuation of therapy should also be assessed [107]. The effectiveness and safety of different bDMARDs and tsDMARDs in PsA should be compared in head–to-head trials or network meta-analyses to enable more evidence-based guidance for treatment selection and sequencing. The feasibility and utility of AI-based decision support tools for individualised therapy selection in PsA patients should be evaluated on the basis of real-world data. In addition to all measurable evidence, patient preferences and values should never be overlooked when choosing a treatment.

It is encouraging to observe that since our data search in October 2023, new and confirming data on the discussed predictors have appeared [108, 109]. Hopefully, genetic and serological markers will also be researched more widely in the future.

## Conclusion

This systematic review and meta-analysis, despite certain limitations, provides valuable insights into the prediction of treatment response to bDMARDs and tsDMARDs in PsA patients. The synthesis of evidence points to several consistent trends, identifying younger age, lower baseline DAPSA, lower baseline HAQ, lower baseline TJC, higher baseline CRP, and male sex as potential predictors of better treatment response. AI has the potential to integrate these and additional predictors that prove to be significant in subsequent research studies into decision support tools for everyday clinical practice and thus make them usable for personalised treatment decisions. A more differentiated therapy design is essential to keep the time with a suboptimal therapy response as short as possible and thereby minimise the long-term consequences of the disease. This review emphasises the need for further studies in this field, and we hope that our findings will guide future research towards, with the aid of AI, the development of individualised treatment strategies for PsA.

### Limitations and strengths

For the interpretation of the results, several strengths and limitations must be considered. This review was conducted according to the PRISMA approach [28], allowing for accurate reproduction. As no restrictions in terms of year of publication or language were made, a thorough and comprehensive review of the currently literature on this topic could be conducted. The weights of the data points were adjusted to account for multiple reporting, such that each study contributed proportionally to the overall results. This increases the validity and reliability of the conclusions. The lack of prepublication and preregistration of the study protocol must be listed as a weakness. To account for this, suspended methodological decisions, mostly selection criteria, were listed in the protocol, and post hoc adjustments were labelled as such in the publication. The exclusive inclusion of published studies and the lack of gray literature could have introduced publication bias, as studies with significant or positive results are more likely to be published [110]. The presence of publication bias was investigated, and evidence of it was found in some cases. The overall heterogeneity among the studies was high, which could limit the generalisability and comparability of the findings. Part of the data heterogeneity can be explained by the inclusion of multiple outcome measures, drugs and assessment times of treatment response as well as the observational nature of the majority of the studies. In addition, the data used originated from univariate or multivariate regression analyses, and the number and type of variables corrected for in the multivariate models varied. Restricting the outcome measures to composite measures could have led to outcome reporting bias. However, the selection of comparable outcome measures was intended to prevent this precisely. Because precalculated data instead of individual patient data were used for the analyses, the adjustment options for possible confounders and thus the precision of the results were limited. Although the evaluation of the risk of bias for the individual studies was generally low and, in no case, high, there was a risk of bias. To assess the robustness of the findings, sensitivity and subgroup analyses were carried out. As the data for this review predominantly originate from observational studies, the resulting evidence is generally considered low quality according to GRADE [48]. Although RCTs are still regarded as having the highest level of evidence, the advantages of observational studies should be considered. Observational studies are not based on a strictly selected population; they also include patients with comorbidities or concomitant therapies, and they often have longer follow-up durations [34]. This brings them much closer to real-life and routine clinical practice, which can be advantageous for certain research questions, such as individual treatment response.

## Supplementary Information

Below is the link to the electronic supplementary material.

Supplementary file1 (DOCX 164 KB)

Supplementary file2 (DOCX 6179 KB)

## Data Availability

The additional digital content details the search string used to identify articles that were considered for inclusion. All extracted data are available within the full texts or accompanying additional materials of the referenced articles.

## Abbreviations

ABT: Abatacept
ACR: American College of Rheumatology
ACR20/50/70: ACR response criteria
ADA: Adalimumab
AI: Artificial intelligence
APT: Apremilast
BASDAI: Bath Ankylosing Spondylitis Disease Activity Index
bDMARD: Biological DMARD
BMI: Body mass index
BSA: Body surface area
CASPAR: Classification for Psoriatic Arthritis
CoE: Certainty of evidence
CPDAI: Composite Psoriatic Disease Activity Index
CRP: C-reactive protein
csDMARD: Conventional synthetic DMARD
CVD: Cardiovascular disease
CZP: Certolizumab pegol
(c)DAPSA: Clinical Disease Activity in Psoriatic Arthritis
DAS28: Disease Activity Score 28
DLQI: Dermatology Life Quality Index
DMARD: Disease-modifying antirheumatic drug
EGOM: EULAR good/moderate response
EMR: Electronic medical record
ESR: Erythrocyte sedimentation rate
ETA: Etanercept
EULAR: European Alliance of Associations of Rheumatology
FFbH: Funktionsfragebogen Hallover
FiRST: Fibromyalgia Rapid Screening Tool
GOL: Golimumab
GRAPPA: Group of Research and Assessment of Psoriasis and Psoriatic Arthritis
HAQ-DI: Health Assessment Questionnaire—Disability Index
I^2^: Higgins & Thompson’s I^2^ statistic
IBD: Inflammatory bowel disease
IFX: Infliximab
IL: Interleukin
ILi: Interleukin inhibitor
IXE: Ixekizumab
JAKi: Janus kinase inhibitor
LEI: Leeds Enthesitis Index
MDA: Minimal disease activity
ML: Machine learning
NOS: Newcastle Ottawa Scale
NSAID: Nonsteroidal anti-inflammatory drug
OR: Odds ratio
PASDAS: Psoriatic Arthritis Disease Activity Score
PASI: Psoriasis Area and Severity Index
PDE4i: Phosphodiesterase-4 inhibitor
PGA: Patient global assessment
PhGA: Physician global assessment
PI: Prediction interval
PRISMA: Preferred Reporting Items for Systematic Reviews and Meta-Analysis
PRO: Patient-reported outcome
PsA: Psoriatic arthritis
PsAID-12: Psoriatic Arthritis Impact of Disease 12-item questionnaire
PsARC: Psoriatic Arthritis Response Criteria
QoL: Quality of life
RA: Rheumatoid arthritis
RCT: Randomised controlled trial
RoB 2: Risk of Bias 2
SDAI: Simplified Disease Activity Index for Rheumatoid Arthritis
SEC: Secukinumab
SJC: Swollen joint count
SpA: Spondylarthritis
T2T: Treat-to-target
TJC: Tender joint count
TNFi: Tumour necrosis factor inhibitor
tsDMARD: Targeted synthetic DMARD
UPA: Upadacitinib
UST: Ustekinumab
τ^2^: Tau squared
