# Supplementary material for: Factors predicting treatment response to biological and targeted synthetic disease-modifying antirheumatic drugs in psoriatic arthritis – a systematic review and meta-analysis
Source: Clin Rheumatol. 2024 Oct 28;43(12):3723–46. doi: 10.1007/s10067-024-07193-y (PMC11582271; doi:10.1007/s10067-024-07193-y)
Supplement: Supplementary file 1 — Supplementary file1 (DOCX 164 KB) [file 10067_2024_7193_MOESM1_ESM.docx]

Systematic Review Protocol

Contents

[Administrative information 2](#_Toc167544571)

[Title 2](#_Toc167544572)

[Registration 2](#_Toc167544573)

[Authors 2](#_Toc167544574)

[Contact information 2](#_Toc167544575)

[Contributions 2](#_Toc167544576)

[Amendments 2](#_Toc167544577)

[Support 2](#_Toc167544578)

[Introduction 2](#_Toc167544579)

[Rationale 2](#_Toc167544580)

[Objectives 5](#_Toc167544581)

[Methods 5](#_Toc167544582)

[Eligibility criteria 5](#_Toc167544583)

[Information sources 6](#_Toc167544584)

[Search strategy 6](#_Toc167544585)

[Study records 6](#_Toc167544586)

[Data management 6](#_Toc167544587)

[Selection process 7](#_Toc167544588)

[Data collection process 7](#_Toc167544589)

[Data items 7](#_Toc167544590)

[Outcomes and prioritization 7](#_Toc167544591)

[Risk of bias in individual studies 7](#_Toc167544592)

[Data synthesis 7](#_Toc167544593)

[Meta-bias(es) 7](#_Toc167544594)

[Confidence in cumulative evidence 7](#_Toc167544595)

# Administrative information

## Title

*Factors predicting treatment response to DMARDs in psoriatic arthritis – a systematic review and meta-analysis*

## Registration

The protocol has not been entered in any register. It has not been published.

## Authors

### Contact information

**Tabea Künzler (TK)**

Medical faculty of the University of Zurich, CH-8091 Zurich, Switzerland

Morillonstrasse 36, CH-3007 Bern, Switzerland

Tabea.kuenzler@uzh.ch

**Manuel Bamert (MB)**

Retail value stream, Galenica AG, Untermattweg 8, CH-3027 Bern, Switzerland

[manuel.bamert@galenicare.com](mailto:manuel.bamert@galenicare.com)

**Prof. Dr. med. Haiko Sprott (HS, corresponding author)**

Medical faculty of the University of Zurich, CH-8091 Zurich, Switzerland

Arztpraxis Prof. Sprott, Hottingerstrasse 44, CH-8032 Zurich, Switzerland

[prof.haiko.sprott@hin.ch](mailto:prof.haiko.sprott@hin.ch)

### Contributions

TK is leading protocol development, analyses and publication. TK and MB are first and second

reviewers. All authors will contribute to data interpretation and article drafts.

HS is head of the project and developed the scientific idea to support TK with her doctoral thesis at the University of Zurich.

Amendments

Important protocol amendments post constitution of the protocol will be recorded and included in publication.

## Support

No sources of support or funding were provided for this review.

#

# Introduction

The introduction has been kept detailed in the protocol in order to clarify the background to this work.

## Rationale

**Psoriatic Arthritis PsA**

*Definition*

Psoriatic arthritis (PsA) is a chronic inflammatory musculoskeletal disease associated with the skin disease psoriasis [1]. “In 1964, psoriatic arthritis (PsA) was recognized as a separate disease by the American Rheumatism Association (now the American College of Rheumatology ACR), and is now included as a member of the spondyloarthropathy spectrum” [2]. The usual absence of rheumatoid factor, an important distinction from rheumatoid arthritis, was described early in the initial disease definition by Moll and Wright [2]. Although “Moll and Wright’s description of PsA suggested that the disease was less severe than that seen in RA” [3], “registry data has shown the destructive and progressive nature of the disease; [and that] it has a similar impact on quality of life [QoL] and functional ability as […] rheumatoid arthritis” [2]. In recent years, several studies have additionally shown increased cardiovascular risk and mortality [2, 4, 5].

*Epidemiology*

The prevalence in the general population is about 0.05-0.25% [6]. In patients with psoriasis Alinaghi [7] found a pooled prevalence of 19.7% and up to 24.6% in case of moderate-to-severe skin disease. “The prevalence of PsA was markedly lower in children and adolescents when compared to adults, but equally frequent in both sexes” [7]. So far, only few risk factors could be confirmed, namely obesity, family history of PsA, and injuries or trauma [6]. There is also substantial evidence that psoriatic arthritis has a genetic component, which is stronger and different than in psoriasis [5].

*Characteristics*

PsA shows a heterogeneous presentation [2], with the majority of patients developing skin symptoms first [1] and arthritis about 10 years later [3]. “Wright identified five clinical patterns among patients with PsA: distal predominant pattern, oligoarticular asymmetrical, polyarticular RA-like, spondylitis, and arthritis mutilans” [3]. Oligoarthritis is the most common pattern, while frequencies in general have varied, partly due to changing patterns throughout the disease course [3]. Nevertheless, “it is essential to assess the disease fully, including arthritis, enthesitis, dactylitis, skin/nail disease and axial involvement” [2]. Besides skin/nail involvement, metabolic syndrome and other cardiovascular complications, the most important non-musculoskeletal comorbidities are uveitis and inflammatory bowel disease (IBD) [2].

*Diagnosis*

“Early diagnosis is important to prevent long term functional disability and to ensure optimal management of arthritis and key comorbidities” [2]. Yet psoriatic arthritis remains underdiagnosed [6]. The CASPAR criteria are the most widely used. Their sensitivity and specificity are both 90% or higher in most studies [6] and they are easy and practical to use in daily practise [8].

Once diagnosed, “the number of actively inflamed joints is one of the best quantifiable musculoskeletal disease activity measures” [9]. But still there is no widely agreed-upon definition of disease severity [1] and “capturing disease activity in this multisystem disease remains challenging” [10]. Hackett & Coates [10] emphasized the need and importance of a regular assessment of disease activity. “Even though […] recommendations indicate that the main goal in PsA treatment should be remission in all disease domains, or, alternatively, low or the lowest possible disease activity [minimal disease activity MDA, treat-to-target approach T2T], there is no consensus on a standardized composite index as an outcome measure to define this goal” [11].

*Pathogenesis and treatment options*

With cardiovascular diseases, “40% of PsA patients fulfill the diagnosis for metabolic syndrome” [2], remaining one of the leading causes of mortality, primary prevention efforts to manage modifiable risk factors are much needed [4, 12]. Our growing understanding of the pathogenesis of PsA, such as the relevance of synovial membrane inflammation and key signaling pathways including IL-12, IL-23 and IL-17, helps to find new treatment options [5]. Today, antirheumatic drugs approved for PsA include a continuously growing selection of conventional synthetic disease-modifying antirheumatic drugs csDMARDs (methotrexate, sulfasalazine, leflunomide), biologic bDMARDs (tumor necrosis factor inhibitors TNFi, interleukin IL-12/23 inhibitors, IL-17A-inhibitors) and targeted synthetic tsDMARDs (phosphodiesterase-4 PDE-4 inhibitors, janus kinase JAK inhibitors) [2]. Despite this ongoing development of new drugs, at least 40% of the patients fail to respond or have only partial response [5] and the need for improvement of therapy remains [11]. ”Both nonpharmacologic and pharmacologic treatment can ameliorate PsA symptoms and can occasionally result in disease remission” [1], but overall, evidence for nonpharmacologic therapies is sparse [13]. Non-pharmacological measures include smoking cessation, some form of combination of exercise, physical therapy, occupational therapy, massage therapy and acupuncture, as well as weight loss [1]. Treatment selection among the various options can be challenging due to the heterogeneous presentation and limited comparative head-to-head evidence. This highlights the importance of shared-decision making (SDM) between the health care provider and the patient [1, 13]. To be able to address all disease domains and comorbidities, we should provide a multispecialty and multidisciplinary care [2].

*Treatment strategy*

The treat-to-target approach (T2T) is the current standard strategy of treatment [14]. “Evidence suggests that patients achieving MDA tend to have less joint damage, improved quality of life and work productivity, and reduced atherosclerosis and arterial stiffness over time” [14]. Both the European Alliance of Associations for Rheumatology (EULAR) and the ACR suggest a step up approach with sequential use of therapeutics [2]. In EULAR’s 2019 update non-steroidal anti-inflammatory drugs (NSAID) and local glucocorticoid injections are proposed as initial therapy, in patients with poor prognostic factors and active arthritis a csDMARD should be rapidly initiated [15]. Requirements and recommendations to start biologic bDMARDs differ from country to country and between the EULAR and the ACR [1, 16]. The ACR guidelines recommend TNFi as a first-line DMARD, whereas the EULAR recommendations suggest using csDMARD (methotrexate in particular) first and to then step up to bDMARDs, such as TNFi, if the treatment goal has not been reached [17].

Despite sparse data, methotrexate (MTX) is often given as first-line DMARD [2, 14, 17]. “Anti-TNFs are superior to csDMARDs in improving arthritis, enthesitis, dactylitis, skin and nails. Further, anti-TNFs are also more efficacious in preventing joint destruction and radiographic progression than cDMARDs” [14]. But biologic therapies are more costly, not as broadly accessible, and to date, there is no evidence for inferior long-term outcomes choosing a less aggressive treatment strategy with a short delay in bDMARD initiation [2, 13]. Similarly, comedication with csDMARDs and bDMARDs opposed to drug switching remains controversial [13, 17]. Within bDMARDs, “the choice of the mode of action should reflect non-musculoskeletal manifestations related to PsA; with clinically relevant skin involvement, preference should be given to an IL-17A or IL-17A/F or IL-23 or IL-12/23 inhibitor; with uveitis to an anti-TNF monoclonal antibody; and with IBD to an anti-TNF monoclonal antibody or an IL-23i or IL-12/23i or a JAKi” [16]. PDE-4 inhibition is mainly used in patients with mild PsA in whom the other drugs are inappropriate [15]. Both PDE-4 and JAK inhibitors (tsDMARDs) have the advantage of possible oral administration [14].

*Treatment response and individualization of therapy*

“Clinical response to drugs varies widely between individuals. A part of this variability is due to the characteristics of the patient such as age, gender, concomitant therapies, body mass index, or smoking status. Clinical response also depends on disease characteristics including disease activity and severity and presence of autoantibodies. Genetic background, cytokine levels, and immune cell phenotypes could also influence biological therapy response” [18]. “Given the heterogeneity of disease presentation of PsA, individualization of therapy is crucial” [12]. Despite promising potential of precision medicine in PsA, currently there is no biomarker to predict treatment response [14]. Most likely no single parameter but panels of biomarkers [10] will guide treatment choice in the future. Further research should incorporate clinical and biological data to develop prediction scores, considering the 3 axes (drug concentrations, disease state, disease pathophysiology) of interindividual variability [18]. “The current practice relies on individualising choice of treatment by matching the most severely affected domains [peripheral joint count, dactylitis, enthesitis, skin, axial disease] of the patients with the best available evidence of efficacies of drugs for those domains” [14]. Cardiovascular risk factors and risks of immunomodulatory therapy should also be considered in the choice of treatment [12]. “When patients do not respond to a treatment, shifting and cycling through different options would be the rational steps” [14]. Reliable predictors could optimize the treatment selection and minimize the time with suboptimal response, thereby reducing the risk for further damage and end organ complications [14].

**Artificial intelligence**

Artificial intelligence (AI) has been used to study rheumatological diseases only for a few years, but machine learning (ML) enables the analysis of large amounts of clinical data and already shows abilities to predict treatment response in other medical fields and even for RA (Kim & Tagkopoulos, 2019; Squarcina et al., 2021). Supervised learning, where machines learn from labeled training data to predict outcome such as treatment response, is the most frequently used method in medical research (Sachiko & Tadahiro, 2022).

## Objectives

The aim of this work is to identify factors predicting treatment response that will enable the development of a decision tool for individual therapy selection in psoriatic arthritis in daily clinical practice. To reach this goal, we want to incorporate epidemiological, biological and clinical data into prediction scores of treatment response to bDMARDs and tsDMARDs in patients with psoriatic arthritis.

Additionally, we will address the capabilities of artificial medicine in medical practice, as well as why and how it may play a key role in our specific research question in the future.

The stated objectives are to be achieved by conducting a systematic literature review with meta-analysis.

# Methods

## Eligibility criteria

- Inclusion criteria:
  - Study types: RCTs, case-control studies, cohort studies
  - Predictors: genetic, biochemical, anatomical, physiological, epidemiological, clinical, radiological, pretreatment, environmental – *exact selection to be defined post-hoc*
  - Therapy: bDMARDs, tsDMARDs
  - Outcomes: quantifiable, composite vs single domain outcome measures, patient-reported – *to be defined post-hoc*
  - Full-text available
  - Human study
  - Adults
  - Data format – *to be defined post-hoc*
- Exclusion criteria:
  - Therapy: only non-pharmalogical, only csDMARDs
  - Study types: case-reports, expert opinion/commentary, review/meta-analysis
  - only other diseases than PsA
  - Predictors: monitoring of treatment response instead of baseline markers, development of disease rather than treatment response
  - Outcomes: drug survival

Studies were excluded if they a) examined non-pharmacological therapies or only csDMARDs, b) focused on factors influencing the development of the disease rather than the treatment response, c) were case-studies, expert opinion or reviews, d) addressed only psoriasis, d) described monitoring biomarkers measured at a later time point than baseline, e) included only radiological, genetic, synovial, skin, dose/concomitant therapy/drug selection or environmental factors, f) used only single domain measures, patient-reported outcomes (PRO) or drug survival as outcome measures, g) addressed only the axial disease axPsA, h) included only PsA-unspecific data of mixed spondyloarthritis (SpA) cohorts. The majority of studies reported the strength of predictors as OR, therefore ORs were used as effect size measure in the statistical analysis. Studies reporting statistically incompatible data were excluded from the meta-analysis.

Patients meeting 5 or more of 7 criteria (tender joint count (TJC), swollen joint count (SJC), enthesitis count, Psoriasis Area and Severity Index (PASI) or body surface area (BSA), patient global assessment (PGA), patient pain, and Health Assessment Questionnaire (HAQ)) are considered to have achieved MDA [19]. The CPDAI assesses disease activity as well as the impact on patient functionality and QoL for 5 PsA domains with scores between 0 and 15 [19]. The DAPSA includes TJC and SJC, patient pain, and global assessments, as well as a C-reactive protein (CRP) measurement [19]. DAS28 comprises the TJC and SJC plus the erythrocyte sedimentation rate (ESR) or CRP level [19]. EULAR response criteria are based on the DAS28 index and categorised by minimum changes and/or thresholds reached [19]. The ACR response criteria require minimal improvements of 20%, 50% or 70% in the TJC and SJC, as do three of the following additional criteria: PGA, patient pain assessment, physician global assessment (PhGA), HAQ score, and ESR or CRP [19]."

To account for multiple reporting of the same study about the strength of a predictor across multiple time points and by using multiple composite measures, the weights of those individual reported outcome measures were adjusted to sum up to 1 for each study. Within the generated forest plots, the studies were sorted according to the time at which the treatment response was assessed.

## Information sources

Electronic databases (from inception): Ovid MEDLINE, Embase, Cochrane Library, Scopus, Web of Science

There will be no language or geographical restrictions.

## Search strategy

All studies that were a) available in full-text, b) randomised controlled trials (RCTs), case-control studies or cohort studies, c) human studies, d) included only adults, e) examined bDMARDs or tsDMARDs, f) addressed demographic, clinical or serological baseline markers predicting treatment response in PsA, g) used composite measures as outcome measures, h) contained odds ratios (ORs) or respective raw data to quantify the strength of the suggested predictors were considered to be included in this study.

The search will be performed using a search protocol with 4 concepts (psoriatic arthritis, bDMARDs/ tsDMARDs, treatment response, predictors/influencing factors).

| **Figure 1.** Details an example of searches that will be used: the Embase advanced search 25th October 2023. | |
| --- | --- |
| 1 | 'psoriatic arthritis'/exp OR (((psoriatic OR psoriasis) NEAR/3 arthritis) OR (psa AND arthritis)):ti,ab |
| 2 | 'treatment response'/exp OR ((therap* OR treatment*) NEAR/3 (respons* OR effectiveness OR efficacy OR efficiency OR potency OR effect* OR outcome*)):ti,ab |
| 3 | 'antirheumatic agent'/exp OR ‘Interleukin 12’/exp OR ‘Interleukin 17’/exp OR ‘Interleukin 23’/exp OR 'tumor necrosis factor inhibitor'/exp OR 'Janus kinase inhibitor'/exp OR 'phosphodiesterase inhibitor'/exp OR 'biological treatment'/exp OR ‘biological product’/exp OR ‘biological therapy’/exp OR (‘disease modifying anti-rheumat*’ DMARD* OR bDMARD* OR tsDMARD* OR Adalimumab OR Certolizumab OR Golimumab OR Infliximab OR Tofacitinib OR Baricitinib OR Abatacept OR Rituximab OR Anakinra OR Tocilizumab OR Etanercept OR Ixekizumab OR Secukinumab OR Ustekinumab OR Guselkumab OR biologic* OR biosimilar* OR Interleukin-23 OR IL-23 OR Interleukin-17 OR IL-17 OR Interleukin-12 OR IL-12 OR ‘Tumor Necrosis Factor*’ OR TNF* OR anti-TNF* OR ‘Janus Kinas*’ OR JAK OR Phosphodiesterase OR PDE*):ti,ab |
| 4 | 'biological marker'/exp OR 'predictor variable'/exp OR 'predictive value'/exp OR ‘influencing factor’/exp OR ((biologic* NEAR/3 marker*) OR predict* OR biomarker* OR biometric* OR ((influenc* OR contribut* OR moderat* OR mediat*) NEAR/3 (factor* OR parameter*)) OR predisp* OR susceptib* OR moderator* OR mediator*):ti,ab |
| 5 | ('animal'/exp OR 'invertebrate'/exp OR 'nonhuman'/exp OR 'animal experiment'/exp OR 'animal tissue'/exp OR 'animal model'/exp OR 'plant'/exp OR 'fungus'/exp) NOT ('human'/exp OR 'human tissue'/exp) |
| 6 | #1 AND #2 AND #3 AND #4 |
| 7 | #6 NOT #5 |
| Total | 960 |

##

## Study records

### Data management

Records will be managed through End Note; specific software for managing bibliographies.

### Selection process

The databases will be searched by the first reviewer (TK). Duplicates will be detected automatically by EndNote. Titles and abstracts as well as full-text articles will be assessed for inclusion by TK and MB.

### Data collection process

Using a standardized form, one reviewer (TK) will extract the data. A second reviewer (MB) will independently check the data for consistency and clarity.

## Data items

Data extracted will include the following summary data: title, author, publication year, sample characteristics, sample size, predictors analyzed, drugs administered, outcome measures, and time of outcome measurements to reflect treatment response.

## Outcomes and prioritisation

The need for a consensus on a standardized composite index as an outcome measure is still unmet in psoriatic arthritis. Because of the various outcome measures used in PsA, we cannot know at this point which exact outcome measures will be included in our study and especially in the meta-analysis.

## Risk of bias in individual studies

The Newcastle Ottawa Scale NOS for observational studies and the Risk of Bias 2 RoB2 tool for randomized-controlled trials will be used to assess risk of bias of individual studies. RoB 2 comprises the following domains: randomization process, deviations from the intended interventions, missing outcome data, measurement of the outcome, and selection of the reported result. The completed assessment forms can be found in the Supplementary Tables S1-S2. There was no weighting or exclusion based on the assessment, but it was taken into account for the overall discussion of the results.

## Data synthesis

Publication bias was tested for by generating and visually assessing contour-enhanced forest plots, and by performing the Egger regression (t-value) as well as Begg and Mazumdar correlation (z-value) tests [40]. The calculations were only carried out for predictors with at least 10 data points (K≥10). The results were incorporated into the certainty assessment described below. For those studies with evidence of publication bias, corrected pooled ORs were calculated using the Duval & Tweedie trim and fill method [37]. A predefined dataset with study characteristics was extracted from each included study. It contained title, author, study size, study type, publication year, mean age, mean disease duration, sex shares, country, outcome measures with measurement times used, as well as drugs and predictors analysed. Data for the statistical analysis was extracted as OR of predictor strength with corresponding confidence intervals and p-values. Whenever possible, ORs from multivariate regression analyses were preferred to those from univariate analyses. If precalculated effect size data was not available, both the ORs and confidence intervals were calculated from raw data. Where grouping of outcomes is possible, meta-analysis will be performed using R Studio. If meta-analysis will not be possible, qualitative data will be incorporated in a meta-synthesis.

## Meta-bias(es)

Data heterogeneity will be assessed by several statistics as I². Publication bias will be estimated by visual assessment of forest plots and quantitatively by performing Egger’s regression and Begg’s rank test. Several sensitivity analyses will be performed. Outliers and influential data points will be identified and excluded. Subgroup analyses for outcome measures and drugs used will be carried out.

## Confidence in cumulative evidence

The strength of the overall body of evidence will be assessed using Grading of Recommendations, Assessment, Development and Evaluation (GRADE).

*From: Shamseer L, Moher D, Clarke M, Ghersi D, Liberati A, Petticrew M, Shekelle P, Stewart L, PRISMA-P Group. Preferred reporting items for systematic review and meta-analysis protocols (PRISMA-P) 2015: elaboration and explanation. BMJ. 2015 Jan 2;349(jan02 1):g7647.*

1. Singh, JA, Guyatt, G, Ogdie, A, Gladman, DD, Deal, C, Deodhar, A*, et al.* 2018 American College of Rheumatology/National Psoriasis Foundation Guideline for the Treatment of Psoriatic Arthritis. *J Psoriasis Psoriatic Arthritis*. 2018;4:31-58.

2. Coates, LC, Helliwell, PS. Psoriatic arthritis: state of the art review. *CME Rheumatology*. 2017;17:65-70.

3. Gladman, DD, Antoni, C, Mease, P, Clegg, DO, Nash, P. Psoriatic arthritis: epidemiology, clinical features, course, and outcome. *Ann Rheum Dis*. 2005;64:ii14-7.

4. Colaco, K, Widdifield, J, Luo, J, Rosen, CF, Alhusayen, R, Paterson, JM*, et al.* Trends in mortality and cause-specific mortality among patients with psoriasis and psoriatic arthritis in Ontario, Canada. *J Am Acad Dermatol*. 2021;84:1302-9.

5. Veale, DJ, Fearon, U. The Pathogenesis of Psoriatic Arthritis. *Lancet*. 2018;391:2273-84.

6. Ogdie, A, Weiss, P. The Epidemiology of Psoriatic Arthritis. *Rheum Dis Clin North Am*. 2015;41:545-68.

7. Alinaghi, F, Calov, M, Kristensen, LE, Gladman, DD, Coates, LC. Prevalence of psoriatic arthritis in patients with psoriasis: A systematic review and meta-analysis of observational and clinical studies. *J Am Acad Dermatol*. 2019;80:251-65.

8. Tillett, W, Costa, L, Jadon, D, Wallis, D, Cavill, C, McHugh, J*, et al.* The ClASsification for Psoriatic ARthritis (CASPAR) criteria--a retrospective feasibility, sensitivity, and specificity study. *J Rheumatol*. 2012;39:154-6.

9. Moller, B, Scholz, GA, Amsler, J, Ciurea, A, Micheroli, R, Nissen, MJ*, et al.* Biological disease-modifying anti-rheumatic drugs are equally effective in psoriatic arthritis patients with low and high joint counts. *Rheumatology (Oxford)*. 2023.

10. Hackett, S, Coates, LC. Outcome measures in psoriatic arthritis: Where next? *Musculoskeletal Care*. 2022;20 Suppl 1:S22-S31.

11. Losinska, K, Michelsen, B, Kavanaugh, A, Korkosz, M, Haugeberg, G. Psoriatic arthritis: improvement in outcomes but persistent sex difference - 5-year follow-up study of a Norwegian outpatient clinic population. *Scand J Rheumatol*. 2023;53:1-11.

12. Coates, LC, Soriano, ER, Corp, N, Bertheussen, H, Callis Duffin, K, Campanholo, CB*, et al.* Group for Research and Assessment of Psoriasis and Psoriatic Arthritis (GRAPPA): updated treatment recommendations for psoriatic arthritis 2021. *Nat Rev Rheumatol*. 2022;18:465-79.

13. Leung, YY, Korotaeva, TV, Candia, L, Pedersen, SJ, Bautista Molano, W, Ruderman, EM*, et al.* Management of Peripheral Arthritis in Patients With Psoriatic Arthritis: An Updated Literature Review Informing the 2021 GRAPPA Treatment Recommendations. *J Rheumatol*. 2023;50:119-30.

14. Noviani, M, Feletar, M, Nash, P, Leung, YY. Choosing the right treatment for patients with psoriatic arthritis. *Ther Adv Musculoskelet Dis*. 2020;12:1-17.

15. Gossec, L, Baraliakos, X, Kerschbaumer, A, de Wit, M, McInnes, I, Dougados, M*, et al.* EULAR recommendations for the management of psoriatic arthritis with pharmacological therapies: 2019 update. *Ann Rheum Dis*. 2020;79:700.1-12.

16. Michelsen, B, Ostergaard, M, Nissen, MJ, Ciurea, A, Moller, B, Ornbjerg, LM*, et al.* Differences and similarities between the EULAR/ASAS-EULAR and national recommendations for treatment of patients with psoriatic arthritis and axial spondyloarthritis across Europe. *Lancet Reg*. 2023;33:1-12.

17. Lindstrom, U, Di Giuseppe, D, Delcoigne, B, Glintborg, B, Moller, B, Ciurea, A*, et al.* Effectiveness and treatment retention of TNF inhibitors when used as monotherapy versus comedication with csDMARDs in 15 332 patients with psoriatic arthritis. Data from the EuroSpA collaboration. *Ann Rheum Dis*. 2021;80:1410-8.

18. Daien, CI, Morel, J. Predictive factors of response to biological disease modifying antirheumatic drugs: towards personalized medicine. *Mediators Inflamm*. 2014;2014:386148.

19. MedicineMatters. At a glance: Common scores used in rheumatology 2018 [updated 21 June 2024; cited 20 December 2023]. Available from: <https://rheumatology.medicinematters.com/treatment/patient-reported-outcomes-/at-a-glance-rheumatology-scores/16077860> (20 December 2023, date last accessed)
