# Supplementary material for: Factors predicting treatment response to biological and targeted synthetic disease-modifying antirheumatic drugs in psoriatic arthritis – a systematic review and meta-analysis
Source: Clin Rheumatol. 2024 Oct 28;43(12):3723–46. doi: 10.1007/s10067-024-07193-y (PMC11582271; doi:10.1007/s10067-024-07193-y)
Supplement: Supplementary file 2 — Supplementary file2 (DOCX 6179 KB) [file 10067_2024_7193_MOESM2_ESM.docx]

**Additional Material**

***AM 1****: Systematic Review Protocol*

See separate document.

***AM 2:*** *Search Protocol used to identify Studies for Screening*

**Database*: Medline***

*25th of October 2023: 517 hits*

| **Concept 1**  *Psoriatic*  *arthritis* | exp Arthritis, Psoriatic/ OR (((psoriatic OR psoriasis) adj3 arthritis) OR (psa AND arthritis)).ti,ab. |
| --- | --- |
|  | **AND** |
| **Concept 2**  *Treatment response* | ((therap* or treatment* or Concept 3) adj3 (respons* or effectiveness or efficacy or efficiency or potency or effect* or outcome*)).ti,ab. |
|  | **AND** |
| **Concept 3**  *DMARDs* | exp antirheumatic agents/ or exp Interleukin-23/ or exp Interleukin-17/ or exp Interleukin-12/ or exp Tumor Necrosis Factor Inhibitors/ or exp Phosphodiesterase 4 Inhibitors/ or exp Receptors, Tumor Necrosis Factor/ or exp biological products/ or exp Janus Kinases/ or ("disease modifying anti-rheumat*" or DMARD* or bDMARD* or tsDMARD* or Adalimumab or Certolizumab or Golimumab or Infliximab or Tofacitinib or Baricitinib or Abatacept or Rituximab or Anakinra or Tocilizumab or Etanercept or Ixekizumab or Secukinumab or Ustekinumab or Guselkumab or biologic* or biosimilar* or Interleukin-23 or IL-23 or Interleukin-17 or IL-17 or Interleukin-12 or IL-12 or Tumor Necrosis Factor* or TNF* or anti-TNF* or Janus Kinas* or JAK or Phosphodiesterase or PDE*).ti,ab. |
|  | **AND** |
| **Concept 4**  *Predictor* | exp Biomarkers/ OR (biomarker* OR biometric* OR predict* OR (biologic* adj3 marker*) OR ((influenc* OR contribut* OR impact* OR moderat* OR mediat*) adj3 (factor* OR parameter*)) OR predisp* OR susceptib* OR moderator* OR mediator*).ti,ab. |
|  | **NOT** |
| **Concept 5**  *Humans* | exp animals/ NOT humans.sh. |

Limits ☒ human ☐ animal

☒ adult ☐ child

☒ all languages ☐ English ☐ German

☐ years ☐ French ☐ Spanish

☐ Italian ☐ other

Database-specific syntax:

Field search Medline .ti,ab.

Proximity Medline adjX

**Database*: EMBASE with Conference Abstracts***

*25th of October 2023: 960 hits*

| **Concept 1**  *Psoriatic*  *arthritis* | 'psoriatic arthritis'/exp OR (((psoriatic OR psoriasis) NEAR/3 arthritis) OR (psa AND arthritis)):ti,ab |
| --- | --- |
|  | **AND** |
| **Concept 2**  *Treatment response* | 'treatment response'/exp OR ((therap* OR treatment*) NEAR/3 (respons* OR effectiveness OR efficacy OR efficiency OR potency OR effect* OR outcome*)):ti,ab |
|  | **AND** |
| **Concept 3**  *DMARDs* | 'antirheumatic agent'/exp OR ‘Interleukin 12’/exp OR ‘Interleukin 17’/exp OR ‘Interleukin 23’/exp OR 'tumor necrosis factor inhibitor'/exp OR 'Janus kinase inhibitor'/exp OR 'phosphodiesterase inhibitor'/exp OR 'biological treatment'/exp OR ‘biological product’/exp OR ‘biological therapy’/exp OR (‘disease modifying anti-rheumat*’ DMARD* OR bDMARD* OR tsDMARD* OR Adalimumab OR Certolizumab OR Golimumab OR Infliximab OR Tofacitinib OR Baricitinib OR Abatacept OR Rituximab OR Anakinra OR Tocilizumab OR Etanercept OR Ixekizumab OR Secukinumab OR Ustekinumab OR Guselkumab OR biologic* OR biosimilar* OR Interleukin-23 OR IL-23 OR Interleukin-17 OR IL-17 OR Interleukin-12 OR IL-12 OR ‘Tumor Necrosis Factor*’ OR TNF* OR anti-TNF* OR ‘Janus Kinas*’ OR JAK OR Phosphodiesterase OR PDE*):ti,ab |
|  | **AND** |
| **Concept 4**  *Predictor* | 'biological marker'/exp OR 'predictor variable'/exp OR 'predictive value'/exp OR ‘influencing factor’/exp OR ((biologic* NEAR/3 marker*) OR predict* OR biomarker* OR biometric* OR ((influenc* OR contribut* OR moderat* OR mediat*) NEAR/3 (factor* OR parameter*)) OR predisp* OR susceptib* OR moderator* OR mediator*):ti,ab |
|  | **NOT** |
| **Concept 5**  *Humans* | ('animal'/exp OR 'invertebrate'/exp OR 'nonhuman'/exp OR 'animal experiment'/exp OR 'animal tissue'/exp OR 'animal model'/exp OR 'plant'/exp OR 'fungus'/exp) NOT ('human'/exp OR 'human tissue'/exp) |

Limits ☒ human ☐ animal

☒ adult ☐ child

☒ all languages ☐ English ☐ German

☐ years ☐ French ☐ Spanish

☐ Italian ☐ other

Database-specific syntax:

Field search EMBASE :ti,ab

Proximity EMBASE NEAR/X

**abase*: Cochrane***

*25^th^ of October 2023:* *113 hits*

| **Concept 1**  *Psoriatic*  *arthritis* | (((psoriatic OR psoriasis) NEAR/3 arthritis) OR (psa AND arthritis)):ti,ab,kw |
| --- | --- |
|  | **AND** |
| **Concept 2**  *Treatment*  *response* | ((therap* OR treatment*) NEAR/3 (respons* OR effectiveness OR efficacy OR efficiency OR potency OR outcome*)):ti,ab,kw |
|  | **AND** |
| **Concept 3**  *DMARDs* | MeSH descriptor: [Antirheumatic Agents] explode all trees OR MeSH descriptor: [Interleukin-12] explode all trees OR MeSH descriptor: [Interleukin-17] explode all trees OR MeSH descriptor: [Interleukin-23] explode all trees OR MeSH descriptor: [Tumor Necrosis Factor Inhibitors] explode all trees OR MeSH descriptor: [Janus Kinase Inhibitors] explode all trees OR MeSH descriptor: [Phosphodiesterase Inhibitors] explode all trees OR MeSH descriptor: [Biological Products] explode all trees OR MeSH descriptor: [Biological Therapy] explode all trees OR ((disease NEXT modifying NEXT anti-rheumat*) OR DMARD* OR bDMARD* OR tsDMARD* OR Adalimumab OR Certolizumab OR Golimumab OR Infliximab OR Tofacitinib OR Baricitinib OR Abatacept OR Rituximab OR Anakinra OR Tocilizumab OR Etanercept OR Ixekizumab OR Secukinumab OR Ustekinumab OR Guselkumab OR biologic* OR biosimilar* OR Interleukin-23 OR IL-23 OR Interleukin-17 OR IL-17 OR Interleukin-12 OR IL-12 OR Tumor Necrosis Factor* OR anti-TNF* OR TNF* OR Janus Kinas* OR JAK OR Phosphodiesterase OR PDE*):ti,ab,kw |
|  | **AND** |
| **Concept 4**  *Predictor* | MeSH descriptor: [Biomarkers] explode all trees OR ((biologic* NEAR/3 marker*) OR predict* OR biomarker* OR biometric* OR ((influenc* OR contribut* OR moderat* OR mediat*) NEAR/3 (factor* OR parameter*)) OR predisp* OR susceptib* OR moderator* OR mediator*):ti,ab,kw |
|  | **NOT** |
| **Concept 5**  *Humans* | MeSH descriptor: [Models, Animal] explode all trees OR MeSH descriptor: [Animal Experimentation] explode all trees |

Limits ☒ human ☐ animal

☒ adult ☐ child

☒ all languages ☐ English ☐ German

☐ years ☐ French ☐ Spanish

☐ Italian ☐ other

Database-specific syntax:

Field search Cochrane :ti,ab,kw

Proximity Cochrane NEAR/X

**Database*: Web of Science Core Collection***

*25^th^ of October 2023:* *269 hits*

| **Concept 1**  *Psoriatic*  *arthritis* | TS = (((psoriatic OR psoriasis) NEAR/3 arthritis) OR (psa AND arthritis)) |
| --- | --- |
|  | **AND** |
| **Concept 2**  *Treatment response* | TS = ((therap* OR treatment*) NEAR/3 (respons* OR effectiveness OR efficacy OR efficiency OR potency OR outcome*)) |
|  | **AND** |
| **Concept 3**  *DMARDs* | TS = (“disease modifying anti-rheumat*” OR DMARD* OR bDMARD* OR tsDMARD* OR Adalimumab OR Certolizumab OR Golimumab OR Infliximab OR Tofacitinib OR Baricitinib OR Abatacept OR Rituximab OR Anakinra OR Tocilizumab OR Etanercept OR Ixekizumab OR Secukinumab OR Ustekinumab OR Guselkumab OR biologic* OR biosimilar* OR Interleukin-23 OR IL-23 OR Interleukin-17 OR IL-17 OR Interleukin-12 OR IL-12 OR “Tumor Necrosis Factor*” OR TNF* OR anti-TNF* OR “Janus Kinas*” OR JAK OR Phosphodiesterase OR PDE*) |
|  | **AND** |
| **Concept 4**  *Predictor* | TS = ((biologic* NEAR/3 marker*) OR predict* OR biomarker* OR biometric* OR ((influenc* OR contribut* OR moderat* OR mediat*) NEAR/3 (factor* OR parameter*)) OR predisp* OR susceptib* OR moderator* OR mediator*) |
|  | **AND** |
| **Concept 5**  *Humans* | AK=("population groups" not "animal models") OR (AB=(men or women or patient or female or male or subjects or adult) NOT AK=("animal models")) |

Limits ☒ human ☐ animal

☒ adult ☐ child

☒ all languages ☐ English ☐ German

☐ years ☐ French ☐ Spanish

☐ Italian ☐ other

Database-specific syntax:

Field search Web of Science TS=(...)

Proximity Web of Science NEAR/X

**Database*: Scopus***

*25^th^ of October 2023:* *860 hits*

| **Concept 1**  *Psoriatic*  *arthritis* | TITLE-ABS-KEY(((psoriatic OR psoriasis) W/3 arthritis) OR (psa AND arthritis)) |
| --- | --- |
|  | **AND** |
| **Concept 2**  *Treatment response* | TITLE-ABS-KEY((therap* OR treatment*) W/3 (respons* OR effectiveness OR efficacy OR efficiency OR potency OR outcome*)) |
|  | **AND** |
| **Concept 3**  *DMARDs* | TITLE-ABS-KEY(“disease modifying anti-rheumat*” OR DMARD* OR bDMARD* OR tsDMARD* OR Adalimumab OR Certolizumab OR Golimumab OR Infliximab OR Tofacitinib OR Baricitinib OR Abatacept OR Rituximab OR Anakinra OR Tocilizumab OR Etanercept OR Ixekizumab OR Secukinumab OR Ustekinumab OR Guselkumab OR biologic* OR biosimilar* OR Interleukin-23 OR IL-23 OR Interleukin-17 OR IL-17 OR Interleukin-12 OR IL-12 OR "Tumor Necrosis Factor*" OR TNF* OR anti-TNF* OR "Janus Kinas*" OR JAK OR Phosphodiesterase OR PDE*) |
|  | **AND** |
| **Concept 4**  *Predictor* | TITLE-ABS-KEY((biological W/3 marker*) OR predict* OR biomarker* OR biometric* OR ((influenc* OR contribut* OR moderat* OR mediat*) W/3 (factor* OR parameter*)) OR predisp* OR susceptib* OR moderator* OR mediator*) |
|  | **AND NOT** |
| **Concept 5**  *Humans* | ((INDEXTERMS(animals OR animal)) AND NOT (INDEXTERMS(humans OR human))) |

Limits ☒ human ☐ animal

☒ adult ☐ child

☒ all languages ☐ English ☐ German

☐ years ☐ French ☐ Spanish

☐ Italian ☐ other

Database-specific syntax:

Field search Scopus TITLE-ABS-KEY(....)

Proximity Scopus W/X oder PRE/X

***AM 3:*** *Study* *Risk of Bias Assessment*

Newcastle – Ottawa Quality Assessment Scale for Cohort Studies (GA Wells, 2021)

Note: A study can be awarded a maximum of one star for each numbered item within the Selection and Outcome categories. A maximum of two stars can be given for Comparability.

*Selection*

1) Representativeness of the exposed cohort

a) truly representative of the average PsA patient treated with b-/csDMARD (not only treatment-naïve or with high disease activity) in the community

b) somewhat representative of the average PsA patient treated with b-/csDMARD in the community

c) selected group of PsA patients (e.g. only treatment-naïve)

d) no description of the derivation of the cohort

2) Selection of the non exposed cohort

a) drawn from the same community as the exposed cohort

b) drawn from a different source

c) no description of the derivation of the non exposed cohort

3) Ascertainment of exposure

a) secure record (e.g. clinical assessment, medical record)

b) structured interview

c) written self report

d) no description

4) Demonstration that outcome of interest was not present at start of study

Treatment target not reached before treatment start.

a) yes

b) no

*Comparability*

Either exposed and non-exposed individuals must be matched in the design and/or confounders must be adjusted for in the analysis. Statements of no differences between groups or that differences were not statistically significant are not sufficient for establishing comparability.

1) Comparability of cohorts on the basis of the design or analysis

a) study controls for sex (rated as the most important factor)

b) study controls for any additional factor

*Outcome*

1) Assessment of outcome

a) Independent or blind assessment stated in the paper, or confirmation of the outcome by reference to secure records (medical records, etc.)

b) Record linkage (e.g. identified through ICD codes on database records)

c) Self-report (i.e. no reference to original medical records to confirm the outcome)

d) No description.

2) Objectively assessed composite outcome measures

a) independent blind assessment

b) record linkage

c) self report

d) no description

3) Was follow-up long enough for outcomes to occur

Adequate follow-up period: 3 months (12 weeks) for 50% improvement/EULAR-good-response – 6 months (24 weeks) for remission/MDA (Gossec et al., 2020; Iannone et al., 2019)

a) yes

b) no

4) Adequacy of follow up of cohorts

a) complete follow up - all subjects accounted for

b) subjects lost to follow up unlikely to introduce bias - small number lost (>80% follow-up (Power, 2023), or description provided of those lost)

c) follow-up rate <80% and no description of those lost

d) no statement

|  | **Selection** | | | | **Comparability** | **Outcome** | | |  |
| --- | --- | --- | --- | --- | --- | --- | --- | --- | --- |
| **Study** | **Representativeness of Exposed** | **Selection of Non-Exposed** | **Ascertainment of Exposure** | **Outcome not Initially Present** | **Cohort Comparability** | **Outcome Assessment** | **Follow-up Duration** | **Follow-up Adequacy** | **Total** |
| *Chimenti 2017* | - (naïve) | * | * | - | ** (sex, MS) | * | * | * (description) | 7 |
| *Venerito 2022* | * | * | * | - | * (not sex) | * | * | * (100%) | 7 |
| *Hojgaard 2015* | - (naïve) | * | * | - | ** (sex, TNFi type) | * | * | * (description) | 7 |
| *Iannone 2013* | * | * | * | * (DAS28 ≥3.2) | ** (sex, other) | * | * | * (description) | 9 |
| *Glintborg 2013* | - (naïve) | * | * | - | ** (sex, many) | * | * | * (description) | 7 |
| *Iannone 2020* | - (naïve) | * | * | - | ** (many, sex) | * | * | * (>80%) | 7 |
| *Chimenti 2012* | * | * | * | * | ** (sex, many) | * | * | *(100%) | 9 |
| *Michelsen 2017* | - (naïve) | * | * | - | ** (sex, other) | * | * | - | 6 |
| *Michelsen 2017* | - (naïve) | * | * | - | ** (sex, other) | * | * | - | 6 |
| *Kristensen 2016* | - (naïve for other bDMARDs) | * | * | - | ** | * | * | * (>80%, description) | 7 |
| *Ramonda 2021* | * | * | * | - | ** (sex, other) | * | * | * (description) | 8 |
| *V.d. Bosch 2010* | * | * | * | - | ** (sex, other) | * | * | * (description, >80%) | 8 |
| *Smolen 2021* | * | * | * | - | ** (sex, many) | * | * | - (imputation, no %) | 7 |
| *Saad 2010* | - (naïve) | * | * | - | ** (sex, many) | * | * | * (>80%) | 6 |
| *Hojgaard 2018* | - (naïve) | * | * | - | ** (many, sex) | * | * | - (no information) | 6 |
| *Miyagawa 2022* | * | * | * | - | - | * | * | * (>80%) | 6 |
| *Haddad 2015* | * (naïve) | * | * | * | ** (sex, many) | * | * | * (description) | 8 |
| *Queiro 2022* | * | * | * | - | * (not sex) | * | - | * (>80%, description) | 6 |
| *Perrotta 2016* | * | * | * | * | ** (sex, many) | * | * | * (>80%) | 9 |
| *Behrens 2018* | * | * | * | - | * (not sex) | * | * | * (description) | 7 |
| *Hojgaard 2016* | - (naïve) | * | * | - | ** (sex, TNFi type) | * | * | * (description) | 7 |
| *Hojgaard 2016* | * | * | * | - | ** (sex, other) | * | * | - | 7 |
| *Gratacos 2007* | * | * | * | - | **(sex, many) | * | * | * | 8 |
| *Iervolino 2012* | - (naïve) | * | * | * | ** (sex, many) | * | - | * (description, >80%) | 7 |
| *Carvalho 2017* | - (naïve) | * | * | - | ** (sex, many) | * | * | * (description) | 7 |
| *Vieira-S. 2020* | * | * | * | - | ** (sex, many) | * | * | * (description) | 8 |
| *Perrotta 2020* | * | * | * | * | - | * | * | * (>80%) | 7 |
| *Venerito 2020* | * | * | * | - | * (not sex) | * | * | * (>80%, description) | 7 |
| *Glintborg 2011* | - (naïve) | * | * | - | ** (sex, many) | * | * | * (description) | 7 |
| *Luchetti G. 2023* | * | * | * | * | ** (sex, many) | * | * | * (>80%, description) | 9 |

Additional Table 1: Newcastle Ottawa Scale NOS

Revised Cochrane risk-of-bias tool for randomized trials (RoB 2) (Cochrane Editorial Board, 2021)

See separate documents.

***AM 4:*** *Qualitative Forest Plots of Statistically Significant Predictors*


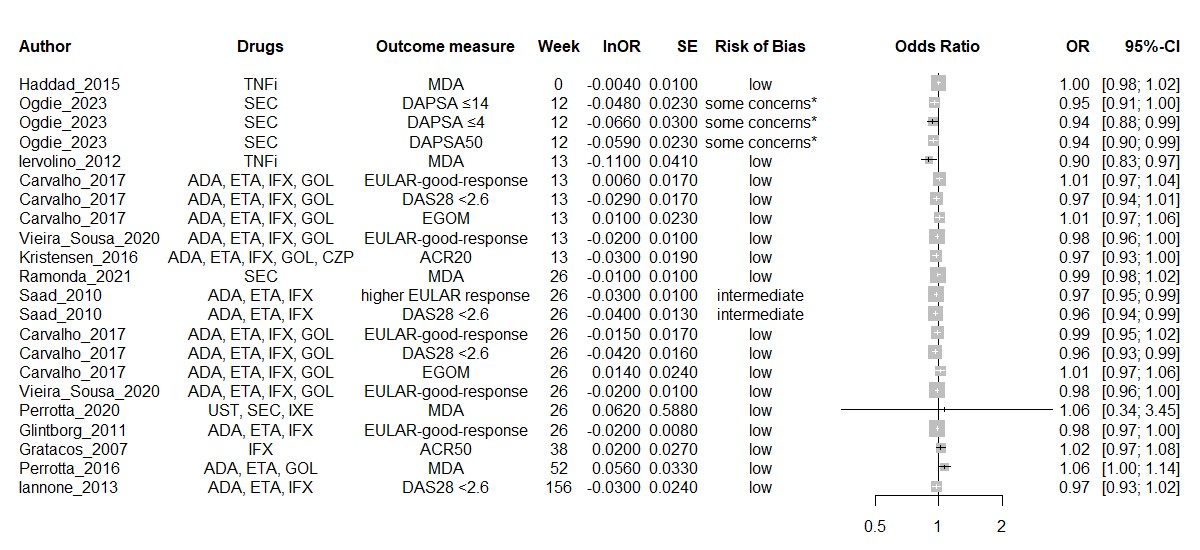


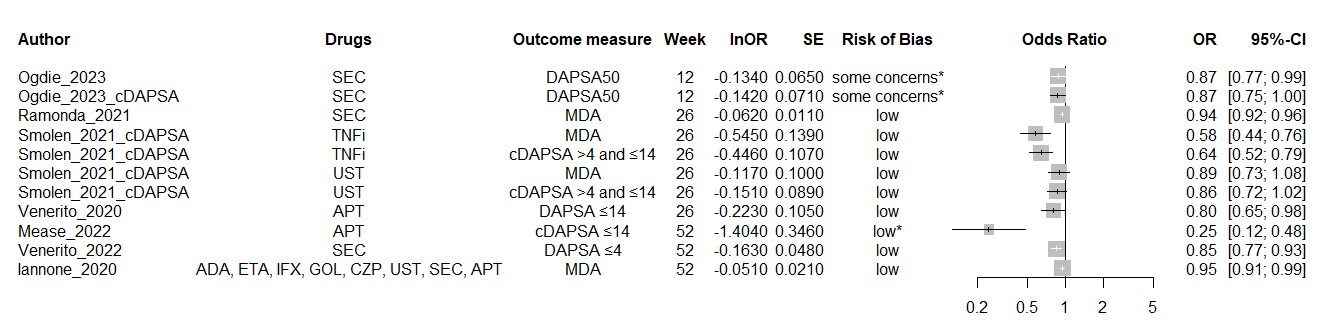


Additional Figure 1: Forest Plot with Original Weights - Age


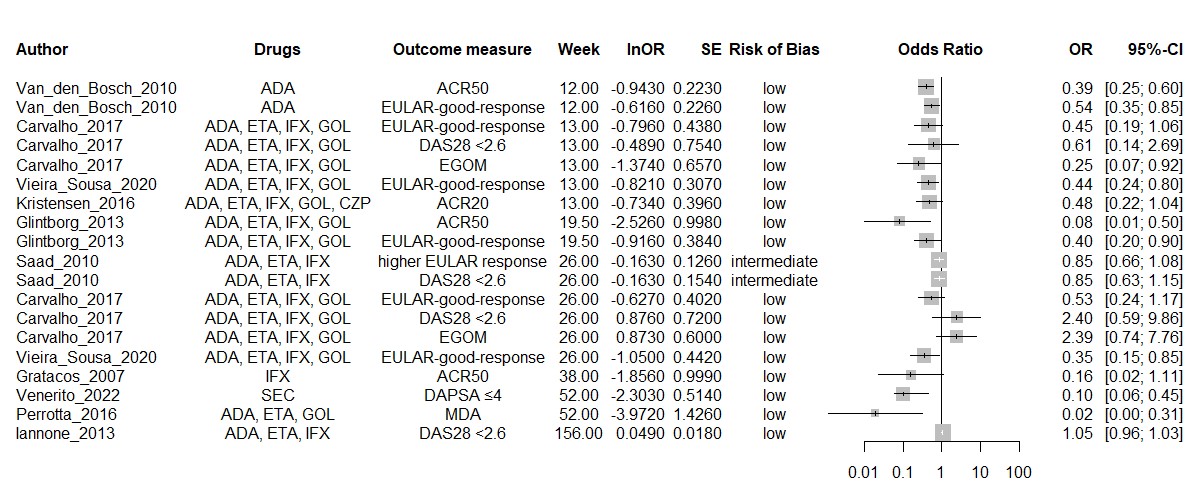


Additional Figure 2: Forest Plot with Original Weights - DAPSA


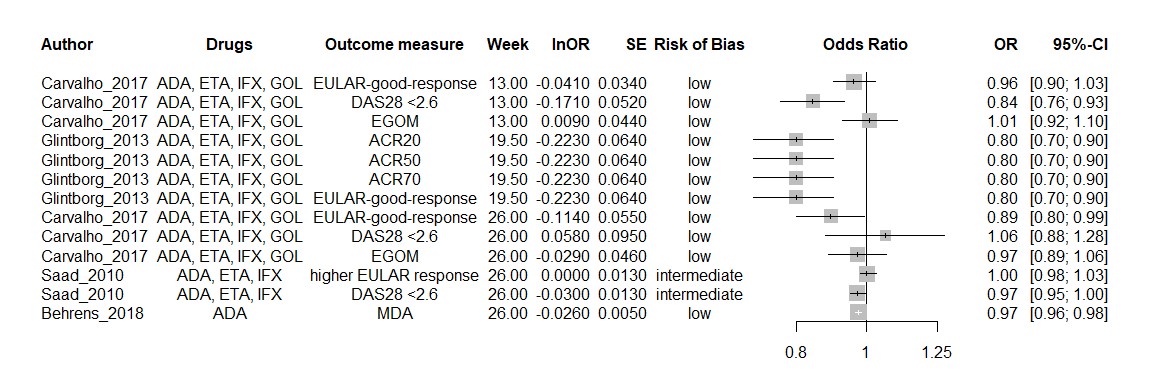


Additional Figure 3: Forest Plot with Original Weights - HAQ


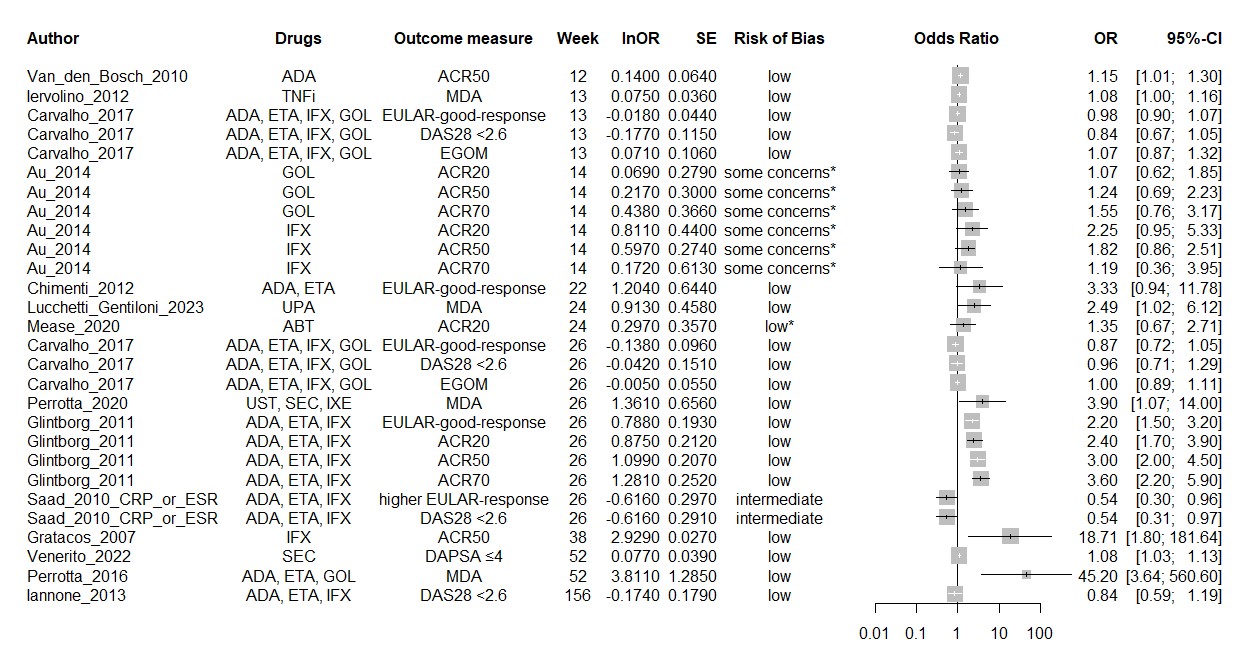


Additional Figure 4: Forest Plot with Original Weights - TJC


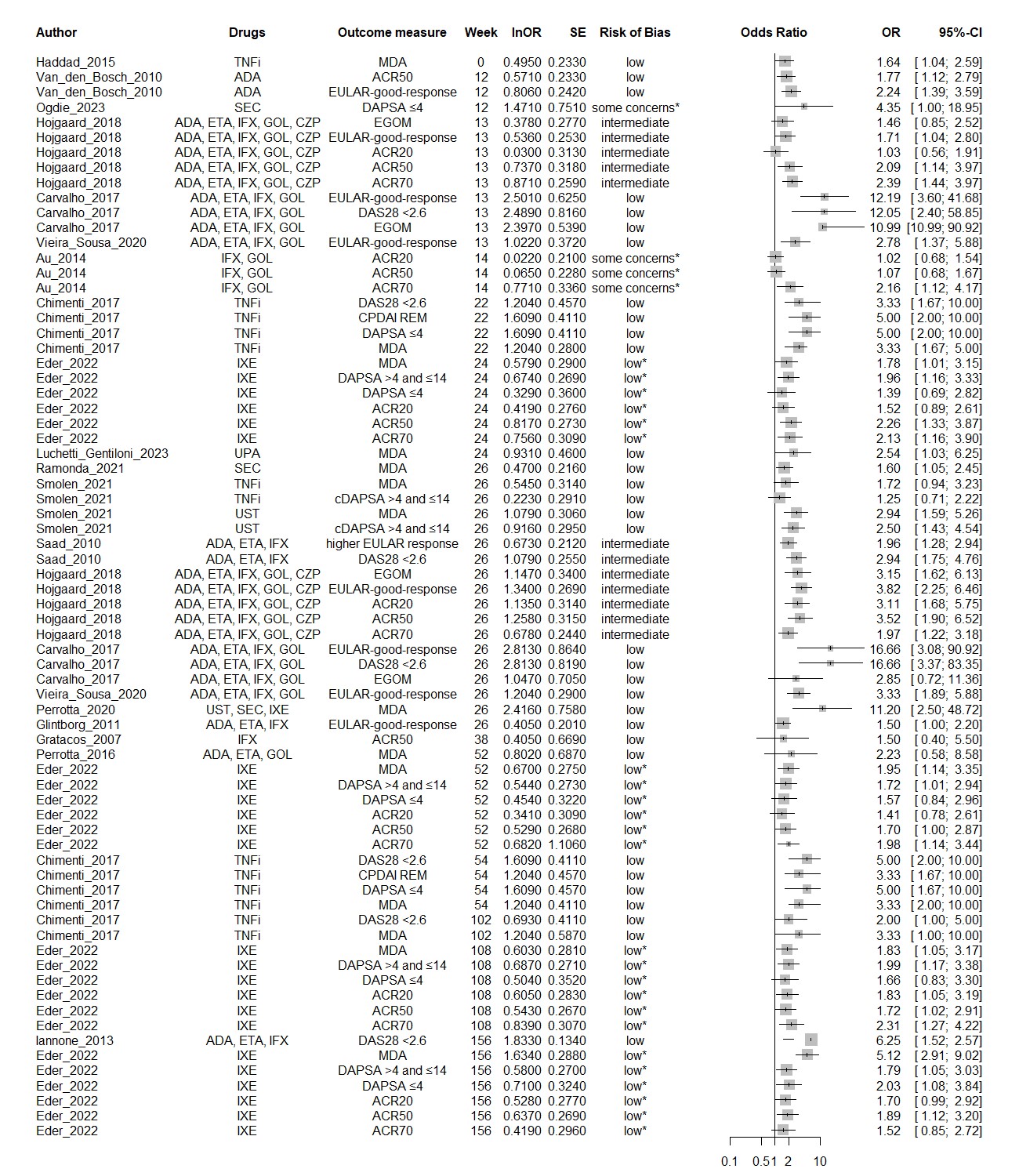


Additional Figure 5: Forest Plot with Original Weights - CRP

***AM 5:*** *Forest Plots of Statistically Non-Significant Predictors*


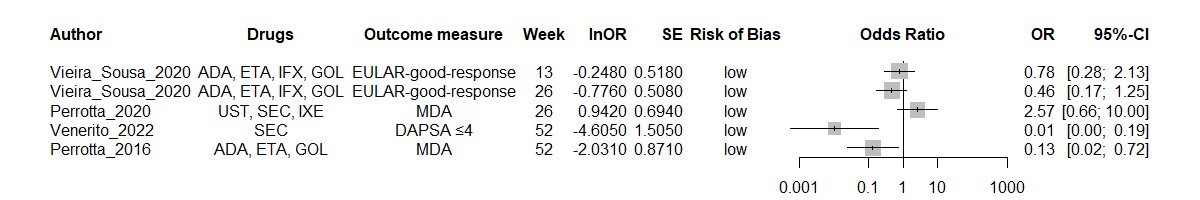


Additional Figure 6: Forest Plot with Original Weights - Male Sex


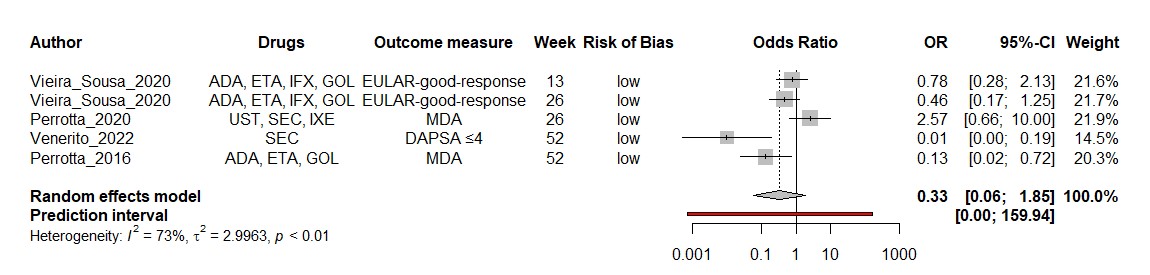


Additional Figure 7: Forest Plot with Original Weights - Axial Disease


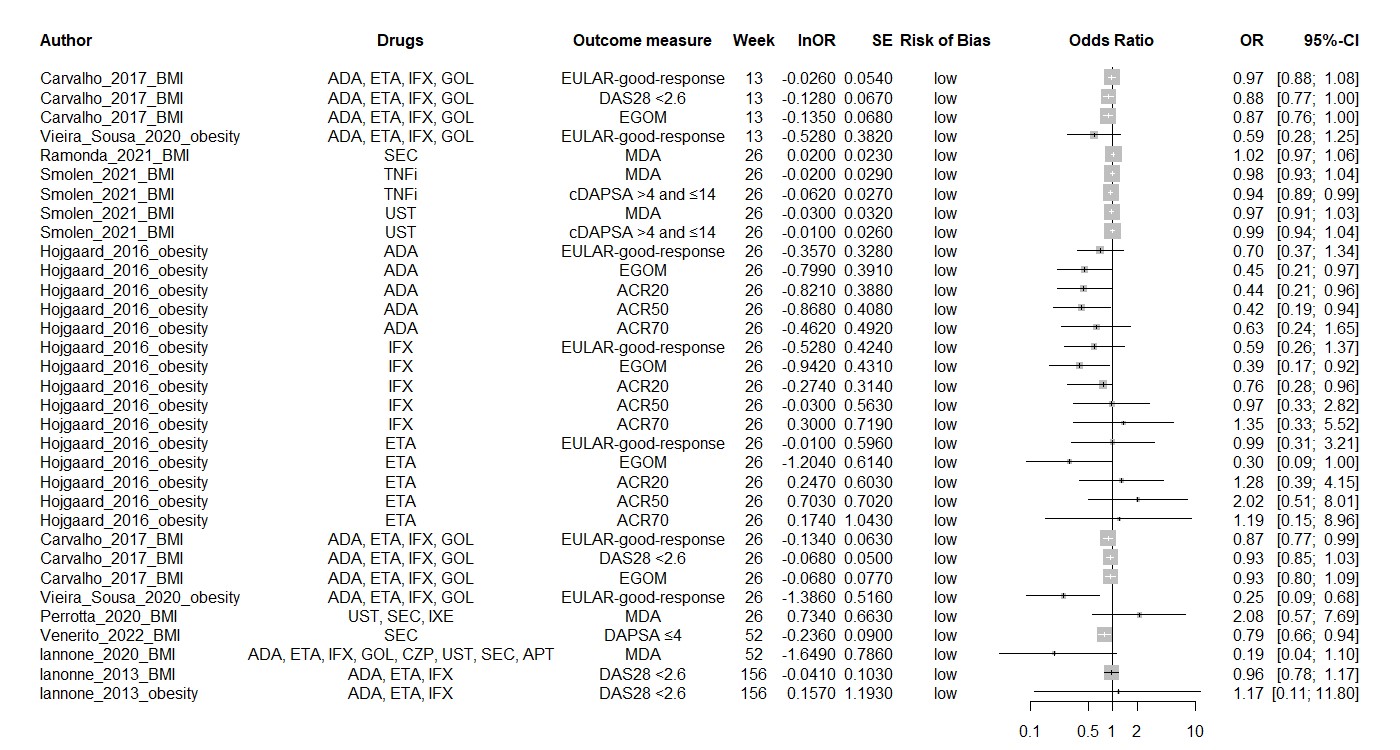


Additional Figure 8: Forest Plot with Adjusted Weights and Pooled OR - Axial Disease


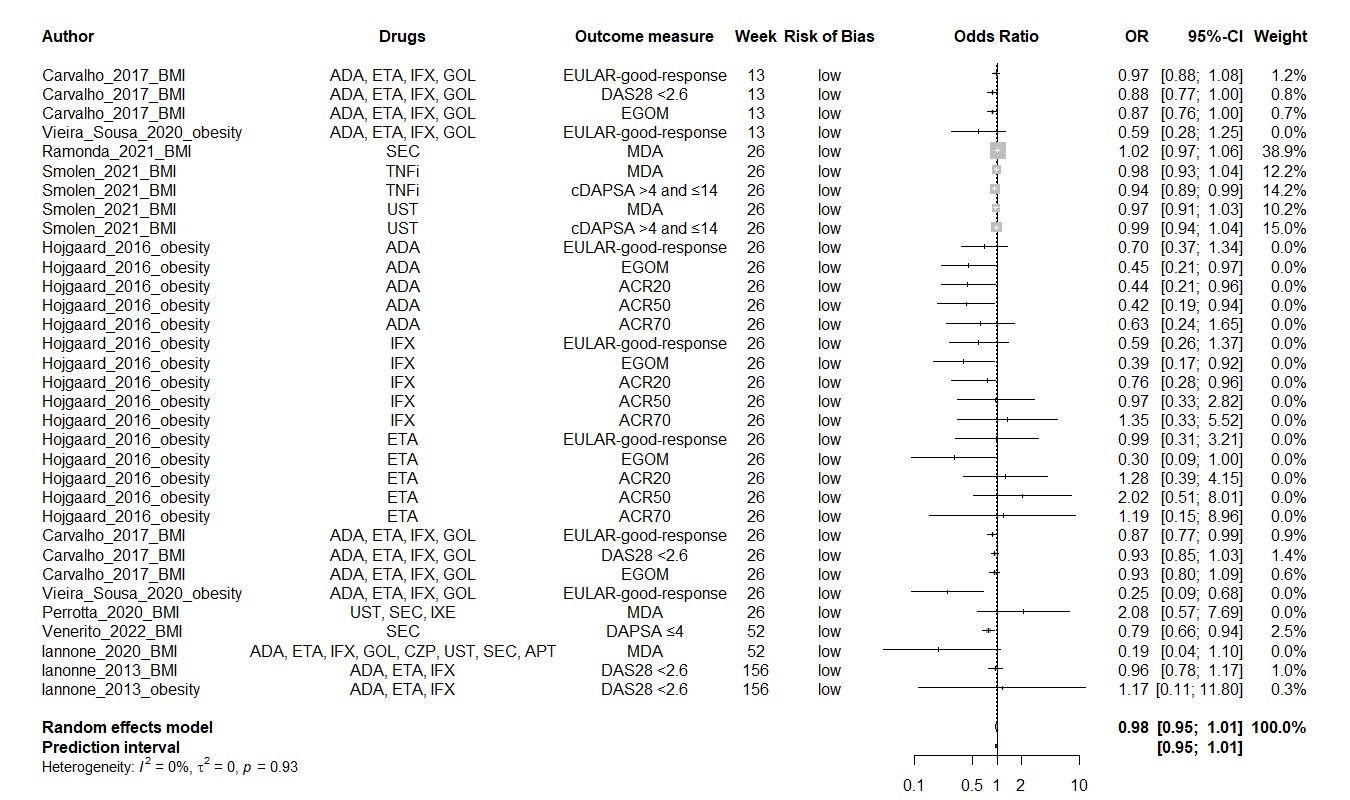


Additional Figure 9: Forest Plot with Original Weights - BMI


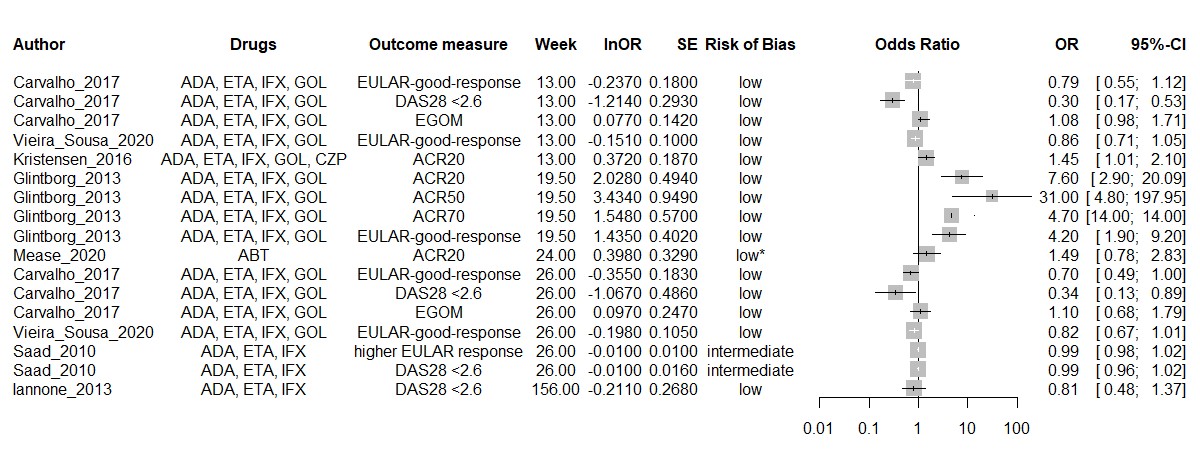


Additional Figure 10: Forest Plot with Adjusted Weights and Pooled OR - BMI


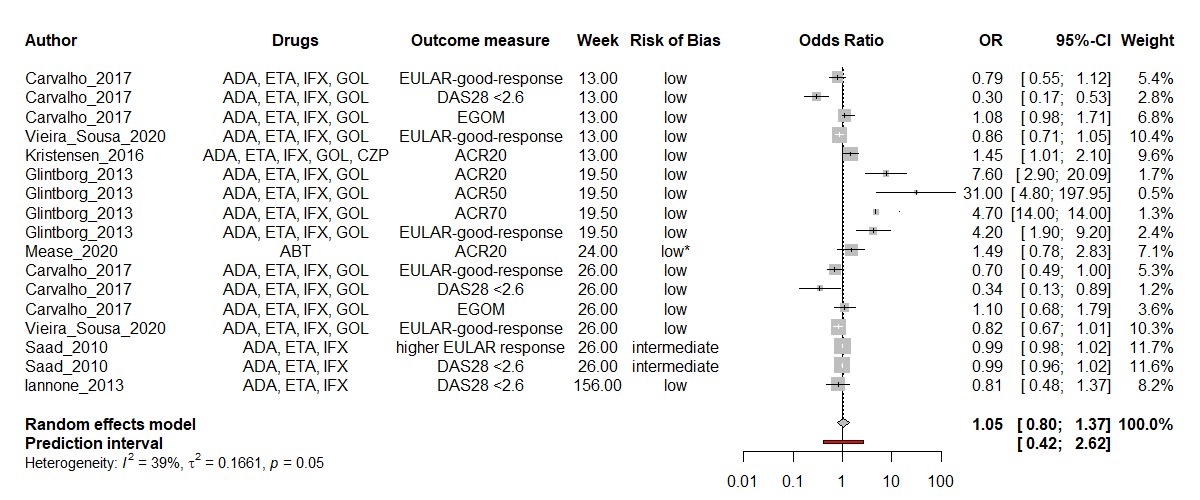


Additional Figure 11: Forest Plot with Original Weights - DAS28


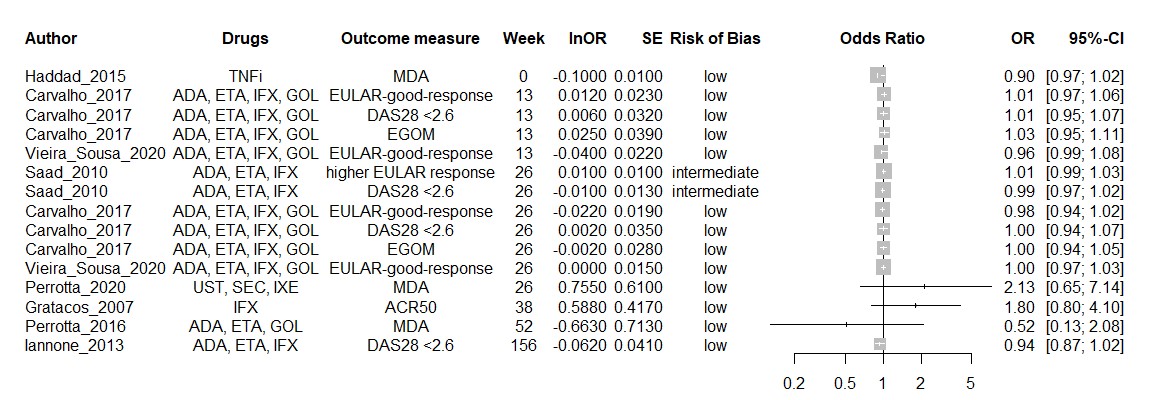


Additional Figure 12: Forest Plot with Adjusted Weights and Pooled OR - DAS28


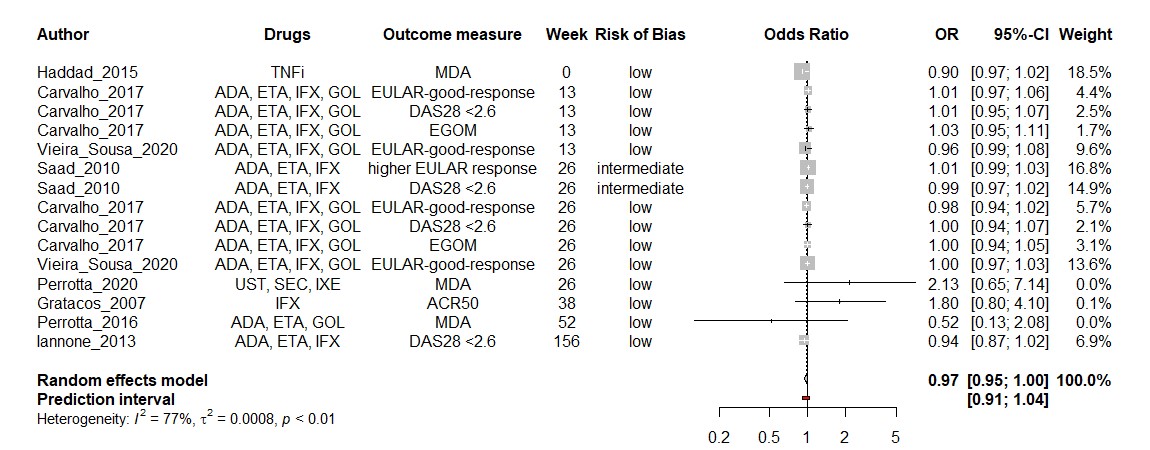


Additional Figure 13: Forest Plot with Original Weights - Disease Duration


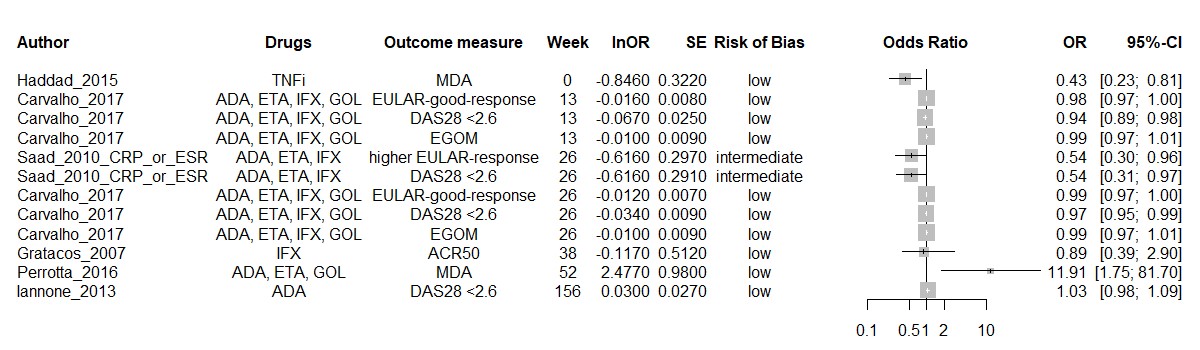


Additional Figure 14: Forest Plot with Adjusted Weights and Pooled OR - Disease Duration


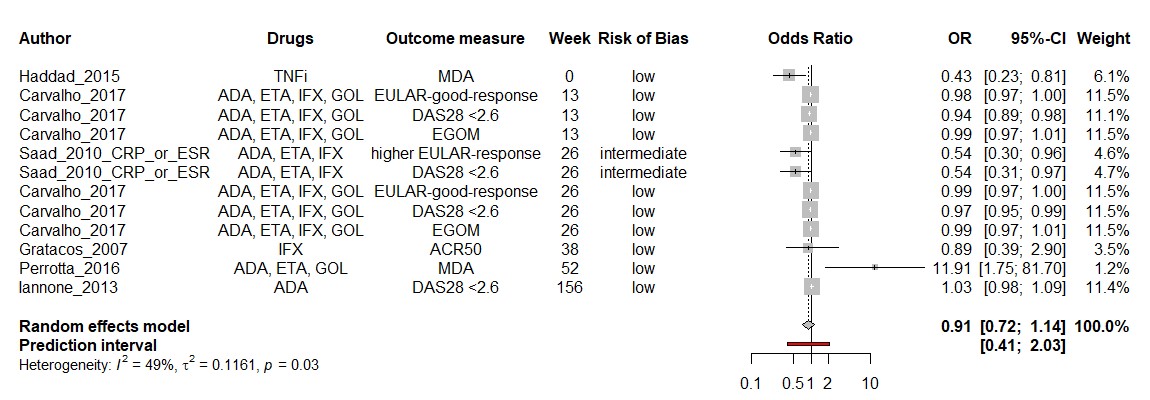


Additional Figure 15: Forest Plot with Original Weights - ESR


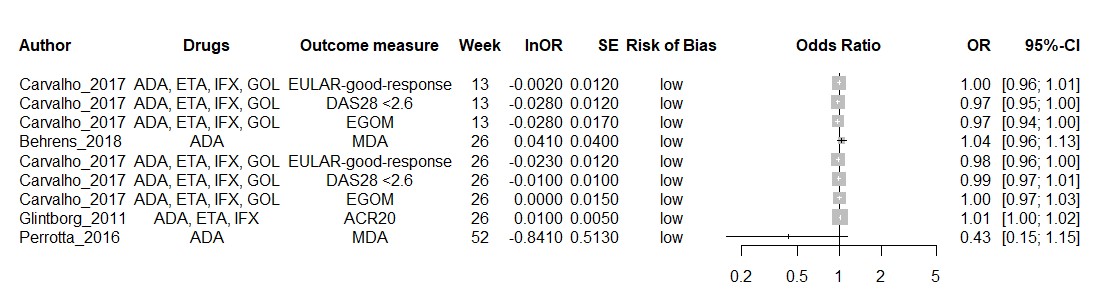


Additional Figure 16: Forest Plot with Adjusted Weights and Pooled OR - ESR


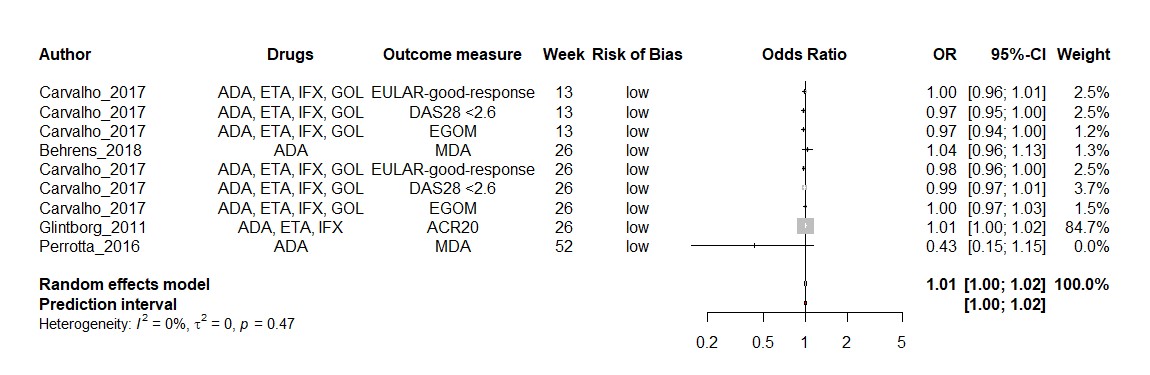


Additional Figure 17: Forest Plot with Original Weights - PGA


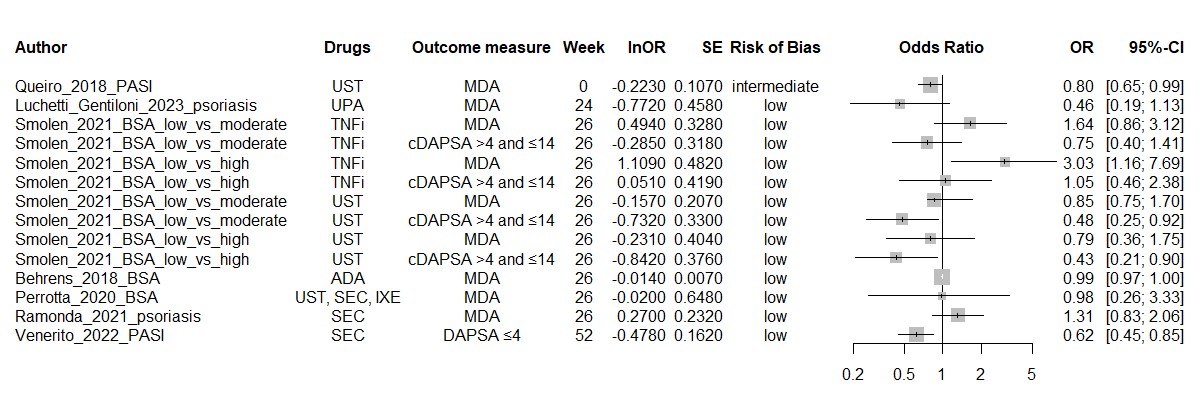


Additional Figure 18: Forest Plot with Adjusted Weights and Pooled OR - PGA


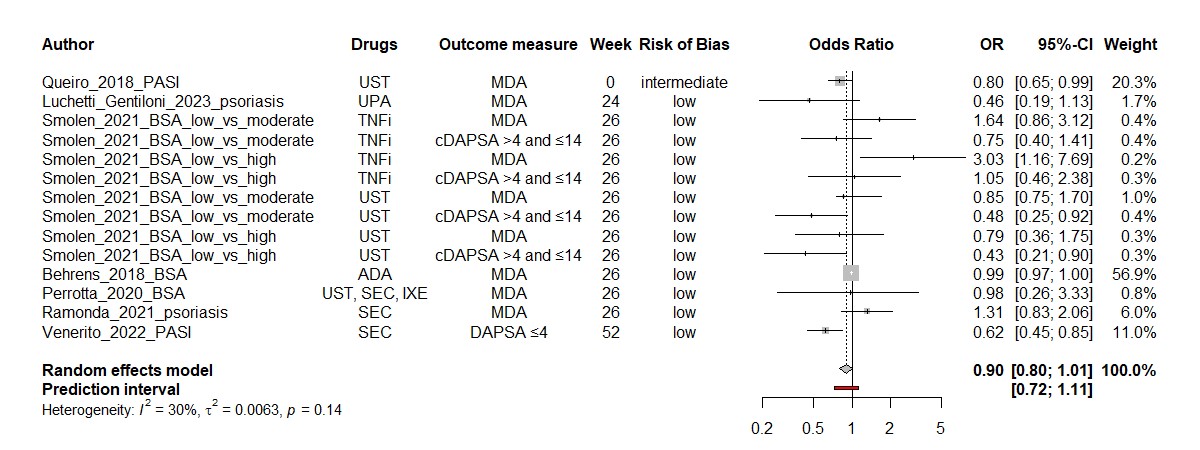


Additional Figure 19: Forest Plot with Original Weights - Psoriasis


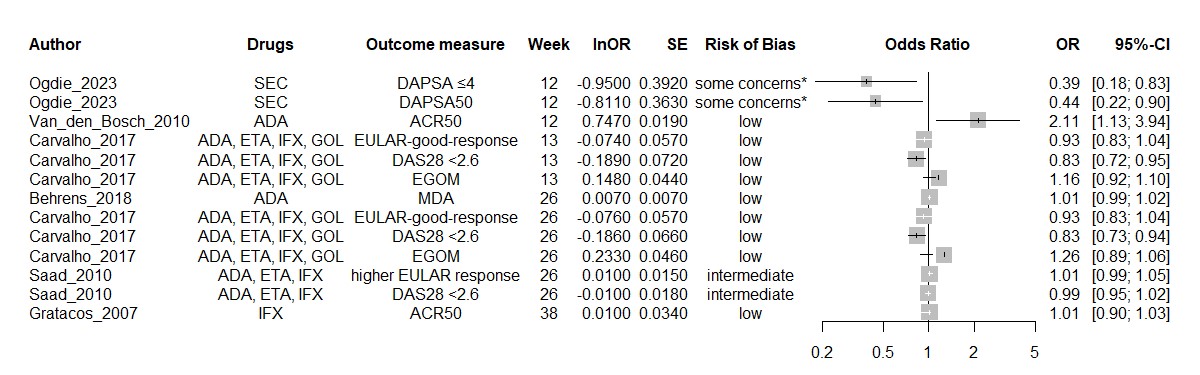


Additional Figure 20: Forest Plot with Adjusted Weights and Pooled OR - Psoriasis


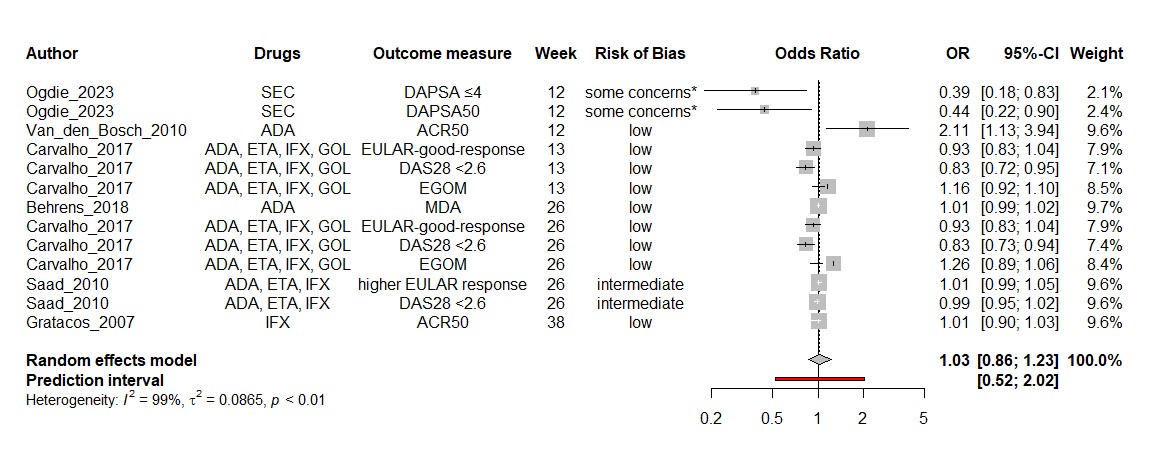


Additional Figure 21: Forest Plot with Original Weights - SJC


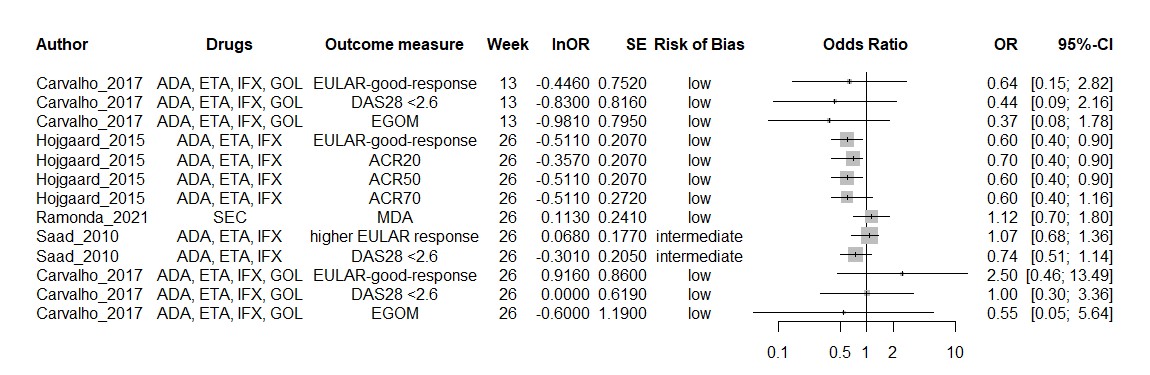


Additional Figure 22: Forest Plot with Adjusted Weights and Pooled OR - SJC


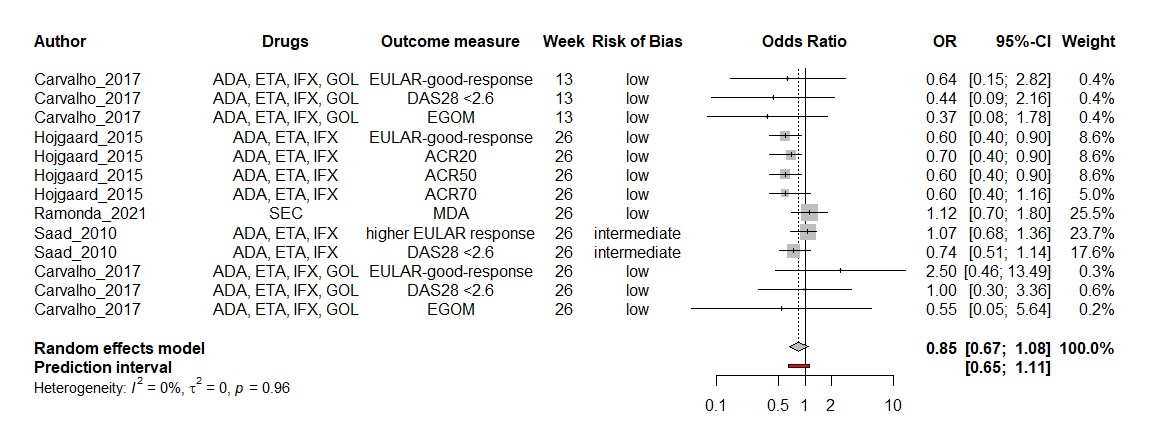


Additional Figure 23: Forest Plot with Original Weights - Smoking


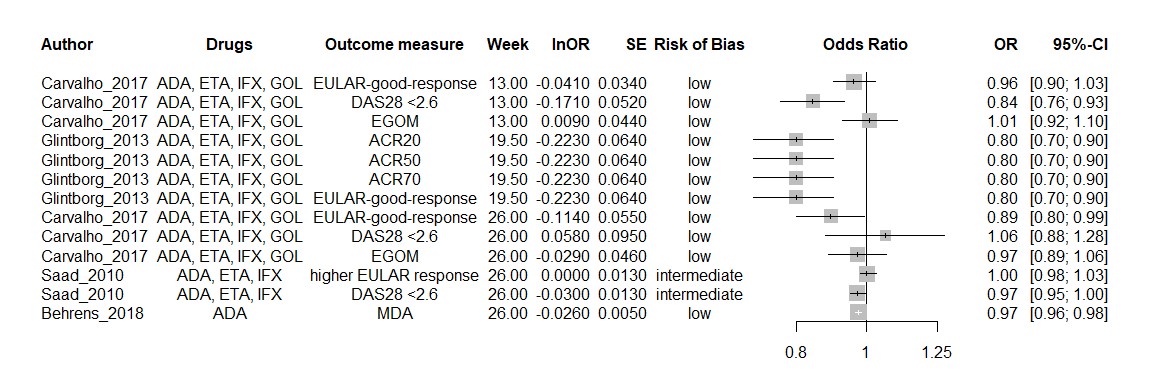


Additional Figure 24: Forest Plot with Adjusted Weights and Pooled OR - Smoking


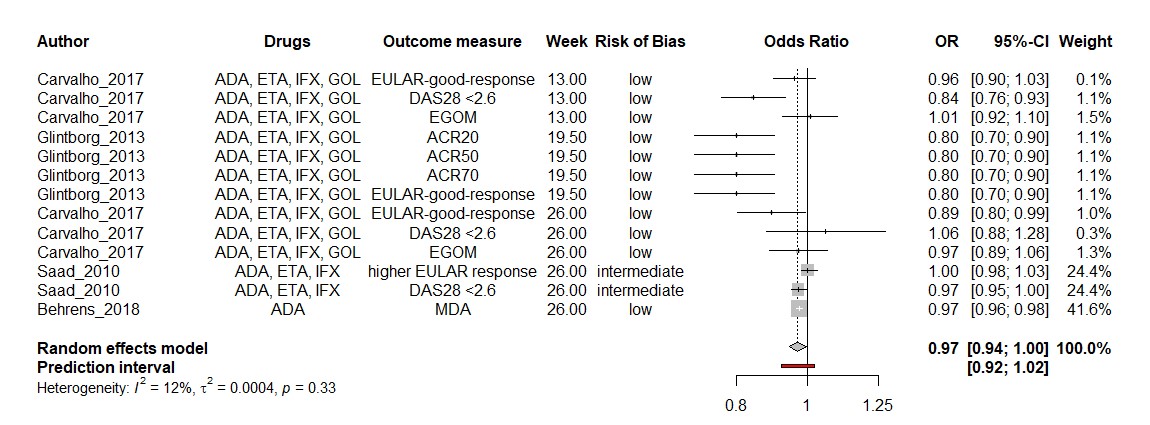


Additional Figure 25: Forest Plot with Original Weights - Treatment Line

***AM 6:*** *Funnel Plots*

Additional Figure 33: Funnel Plot - Axial Disease


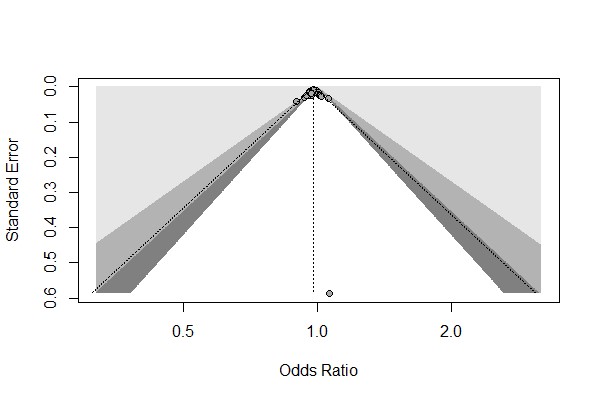

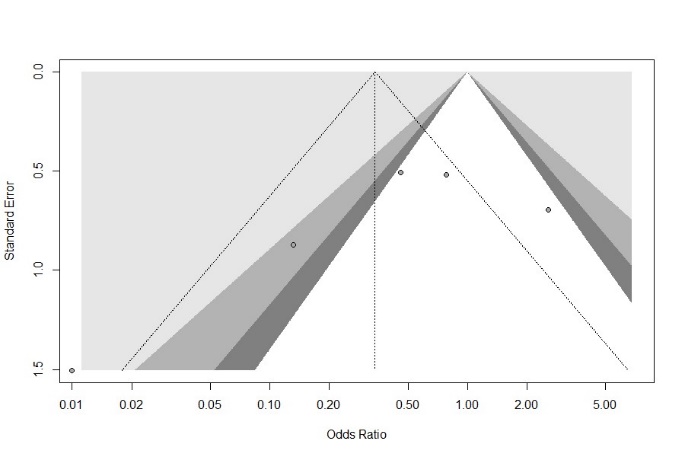


Additional Figure 33: Funnel Plot - Age


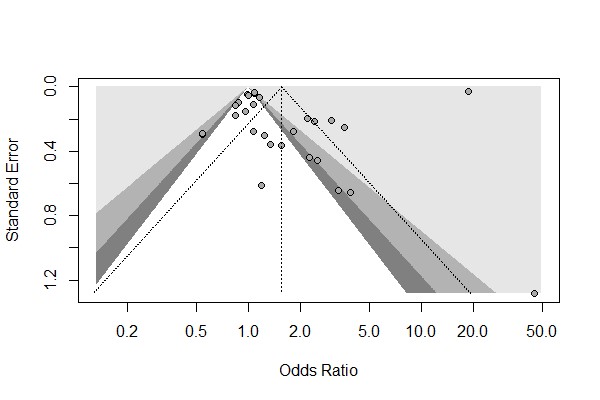

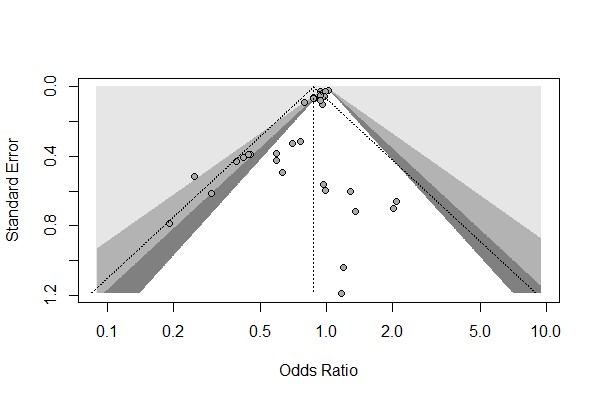


Additional Figure 33: Funnel Plot - CRP

Additional Figure 33: Funnel Plot - DAS28


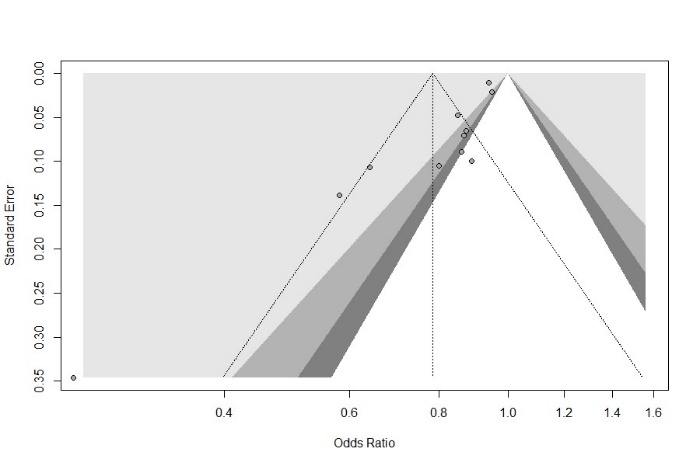

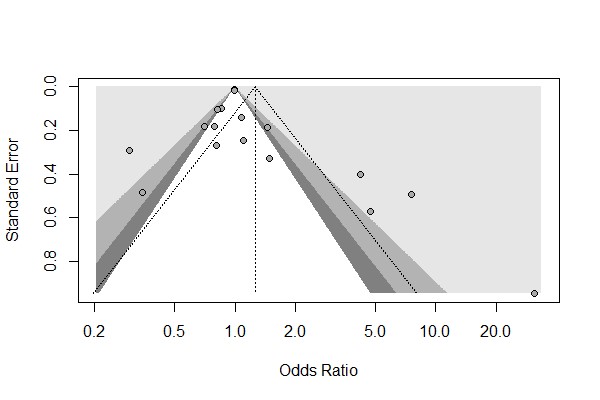


Additional Figure 33: Funnel Plot - DAPSA


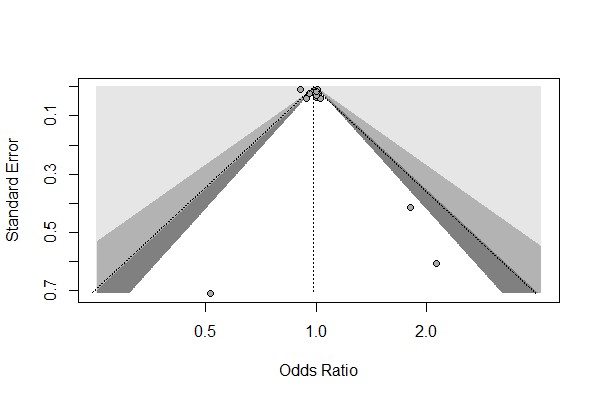

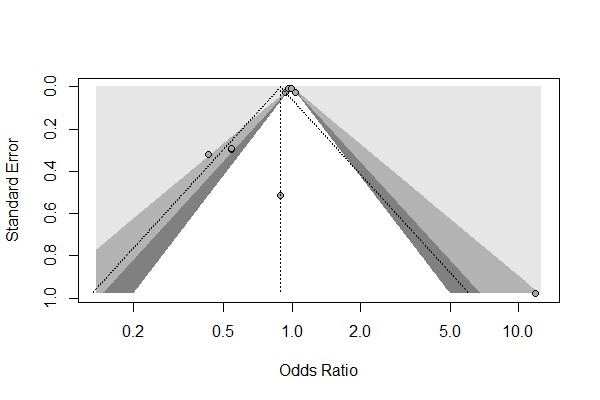


Additional Figure 33: Funnel Plot - Disease Duration

Additional Figure 33: Funnel Plot - ESR

Additional Figure 34: Funnel Plot - BMI


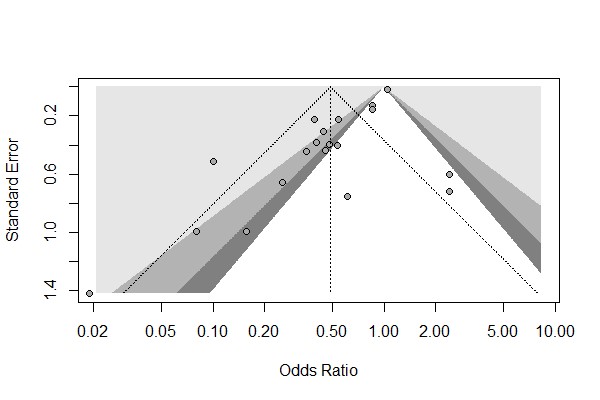

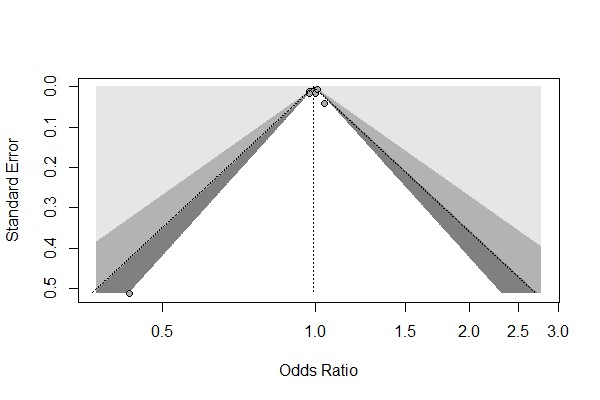


Additional Figure 42: Funnel Plot - HAQ

Additional Figure 42: Funnel Plot - PGA


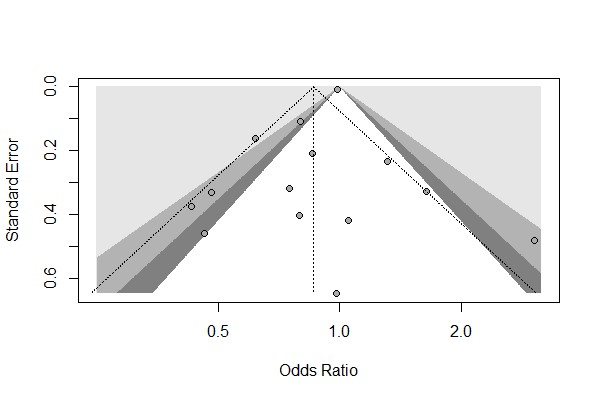

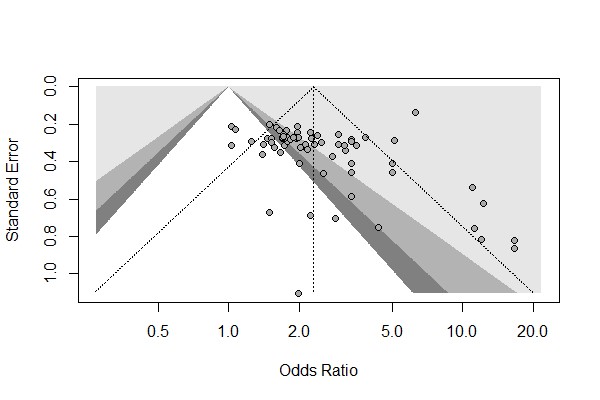


Additional Figure 42: Funnel Plot - Psoriasis

Additional Figure 42: Funnel Plot - Sex


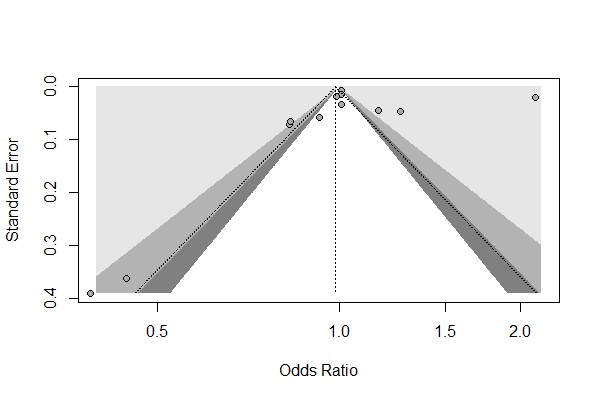

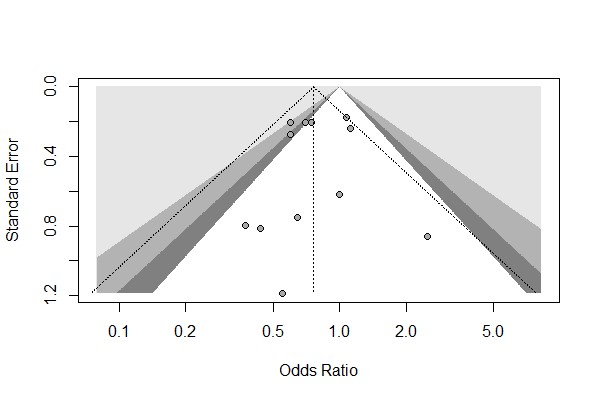


Additional Figure 42: Funnel Plot - SJC

Additional Figure 42: Funnel Plot - Smoking


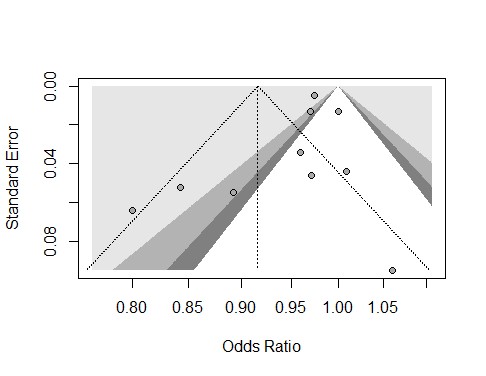


Additional Figure 42: Funnel Plot - TJC


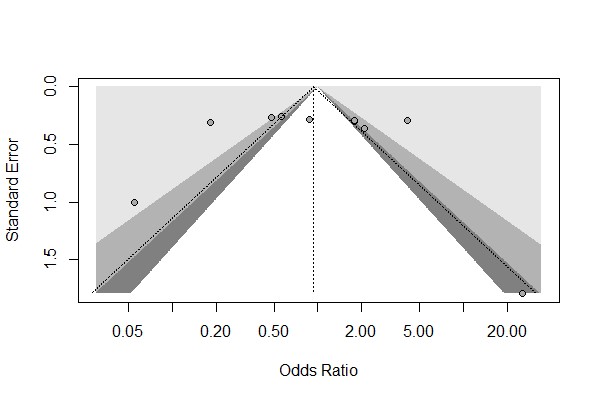


Additional Figure 42: Funnel Plot - Treatment Line

***AM 7****: Certainty of Evidence Assessment – GRADE* (Kirmayr, 2021)

Additional Table 2: GRADE Assessment

| **Certainty Assessment** | | | | | | | | **Effect** | **Certainty** |
| --- | --- | --- | --- | --- | --- | --- | --- | --- | --- |
| *Predictor* | *No. of Studies* | *Study Designs* | *Risk of Bias* | *Inconsistency (I²>50%)* | *Indirectness* | *Imprecision* | *Other*  *considerations* | *OR (95% CI)* |  |
| Age | 13 | Observational studies, (1 RCT) | Not serious | Not serious | Not serious | Not serious | None | 0.982 (0.975-0.99) | ꚚꚚꓳꓳ Low |
| Axial disease | 4 | Observational studies | Not serious | Serious | Not serious | Serious | None | 0.335 (0.06-1.852) | Ꚛꓳꓳꓳ Very low |
| BMI | 9 | Observational studies | Not serious | Not serious | Not serious | Not serious | Publication bias strongely suspected | 0.98 (0.953-1.008) | Ꚛꓳꓳꓳ Very low |
| CRP | 14 | Observational studies, (2 RCT) | Not serious | Serious | Not serious | Not serious | Publication bias suspected | 1.537 (1.111-2.125) | Ꚛꓳꓳꓳ  Very low |
| DAPSA | 7 | 4 Observational studies, 3 RCTs | Not serious | Serious | Not serious | Not serious | Publication bias strongely suspected | 0.789 (0.663-0.938) | Ꚛꓳꓳꓳ  Very low |
| DAS28 | 7 | Observational studies, (1 RCT) | Not serious | Not serious | Not serious | Serious | None | 1.046 (0.796-1.374) | Ꚛꓳꓳꓳ  Very low |
| Disease duration | 8 | Observational studies | Not serious | Serious | Not serious | Not serious | None | 0.974 (0.95-1.0) | Ꚛꓳꓳꓳ  Very low |
| ESR | 6 | Observational studies | Not serious | Not serious | Not serious | Serious | None | 0.909 (0.725-1.14) | Ꚛꓳꓳꓳ  Very low |
| HAQ | 10 | Observational studies | Not serious | Serious | Not serious | Not serious | Publication bias suspected | 0.518 (0.336-0.798) | Ꚛꓳꓳꓳ  Very low |
| PGA | 4 | Observational studies | Not serious | Not serious | Not serious | Not serious | None | 1.007 (0.998-1.016) | ꚚꚚꓳꓳ  Low |
| Psoriasis | 7 | Observational studies | Not serious | Not serious | Not serious | Not serious | None | 0.898 (0.798-1.01) | ꚚꚚꓳꓳ  Low |
| Sex | 18 | Observational studies, (3 RCT) | Not serious | Not serious | Not serious | Not serious | Publication bias suspected | 2.188 (1.912-2.503) | ꚚꚚꓳꓳ  Low |
| Smoking | 4 | Observational studies | Not serious | Not serious | Not serious | Serious | None | 0.853 (0.672-1.082) | Ꚛꓳꓳꓳ  Very low |
| Treatment line | 6 | Observational studies, (1 RCT) | Not serious | Serious | Not serious | Serious | None | 0.935 (0.389-2.249) | Ꚛꓳꓳꓳ  Very low |
| SJC | 6 | Observational studies, (1 RCT) | Not serious | Serious | Not serious | Serious | None | 1.028 (0.859-1.229) | Ꚛꓳꓳꓳ  Very low |
| TJC | 4 | Observational studies | Not serious | Not serious | Not serious | Not serious | Publication bias strongely suspected | 0.97 (0.945-0.996) | Ꚛꓳꓳꓳ  Very low |

*References*

Cochrane Editorial Board. (2021). *Risk of Bias 2 (RoB 2) tool*. Retrieved 17th of December 2023 from <https://methods.cochrane.org/risk-bias-2>

GA Wells, B. S. (2021). *The Newcastle-Ottawa Scale (NOS) for assessing the quality of nonrandomised studies in meta-analyses*. Retrieved 17th of December 2023 from <https://www.ohri.ca/programs/clinical_epidemiology/oxford.asp>

Gossec, L., Baraliakos, X., Kerschbaumer, A., de Wit, M., McInnes, I., Dougados, M., Primdahl, J., McGonagle, D. G., Aletaha, D., Balanescu, A., Balint, P. V., Bertheussen, H., Boehncke, W.-H., Burmester, G. R., Canete, J. D., Damjanov, N. S., Kragstrup, T. W., Kvien, T. K., Landewé, R. B. M., . . . Smolen, J. S. (2020). EULAR recommendations for the management of psoriatic arthritis with pharmacological therapies: 2019 update. *Annals of the rheumatic diseases*, *79*(6), 700.701-712. <https://doi.org/10.1136/annrheumdis-2020-217159>

Iannone, F., Semeraro, A., Carlino, G., Santo, L., Bucci, R., Quarta, L., Maruotti, N., Zuccaro, C., Marsico, A., Falappone, P. C. F., Mazzotta, D., Cantatore, F. P., Muratore, M., & Lapadula, G. (2019). Effectiveness of Certolizumab-Pegol in Rheumatoid Arthritis, Spondyloarthritis, and Psoriatic Arthritis Based on the BIOPURE Registry: Can Early Response Predict Late Outcomes? *Clinical drug investigation*, *39*(6), 565-575. <https://doi.org/https://dx.doi.org/10.1007/s40261-019-00782-9>

Kirmayr, M. (2021). *The GRADE approach, Part 1: how to assess the certainty of the evidence*. Retrieved 17th of December 2023 from <http://viejo.medwave.cl/link.cgi/English/Reviews/MethodlogicalNotes/8110.act>

Power. (2023). *Lost to follow-up: Where did the lost patients go?* Retrieved 17th of December 2023 from <https://www.withpower.com/guides/lost-to-follow-up>
